# Functional foods in Mediterranean diet: exploring the functional features of vegetable case-studies obtained also by biotechnological approaches

**DOI:** 10.1007/s40520-024-02860-1

**Published:** 2024-10-16

**Authors:** Anna Rita Bavaro, Annamaria Tarantini, Angelica Bruno, Antonio F. Logrieco, Antonia Gallo, Giovanni Mita, Francesca Valerio, Gianluca Bleve, Angela Cardinali

**Affiliations:** 1 National Research Council, Institute of Sciences of Food Production (CNR-ISPA), Bari, 70126 Italy; 2 National Research Council, Institute of Sciences of Food Production (CNR-ISPA), Lecce, 73100 Italy; 3 Xianghu Lab, Biomanufactoring Institute, Hangzhou, Zhejiang China

**Keywords:** Polyphenols, Antiaging, Fermentation, Bioaccessibility, Bioavailability

## Abstract

The Mediterranean Diet (MedDiet) is a widely recognized dietary pattern, with its effects largely attributed to “functional foods” which are able to positively influence one or more target functions, improving health and maintaining a state of well-being.

In this review, three “case-study” typical of the MedDiet, such as artichokes, capers and table olives are considered as traditional functional vegetables rich in bioactive compounds, mainly polyphenols. The review extensively discusses the antioxidant effects of these molecules, as well as their role in aging prevention and reduction, maintaining human health, and influencing the abundance and composition of intestinal microbiota. Additionally, this review focuses on the fate of the dietary polyphenols along the digestive tract.

Among biotechnological strategies, the review explores the role of fermentation process in modifying the biochemical profile, recovery, bioaccessibility and bioavailability of bioactive compounds present in some vegetable foods of MedDiet. Finally, the main challenges in the selection, addition, and maintenance of probiotic strains in traditional food products are also summarized, with a view to develop new probiotic carriers for “functional diets”.

## Introduction

The Mediterranean Diet (MedDiet) is defined as a dietary pattern mostly including fresh vegetables and fruit, nuts, daily consumption of non-refined cereals (and derived products), olive oil, moderate amounts of fish, eggs, sweets, poultry and potatoes, monthly consumption of red meat and regular physical activity [1–3]. This dietary pattern has been related to the reduction of coronary heart disease (CHD) risk compared to the United States and the European countries, as stated in the Seven Countries Study (https://www.sevencountriesstudy.com) [4].

The beneficial effects of MedDiet are attributed to “functional foods”, defined as “Foods for Specified Health Use” (FOSHU) due to their ability to beneficially affect one or more target functions, thereby improving health and maintaining well-being by reducing disease risk. A consensus document was established within the FUFOSE-PASSCLAIM project [5] and regulated by Japanese Law since 1991. The functional effect of a specific food is related to the presence of metabolites, dietary fiber, and active ingredients that exert a beneficial impact on human health. The health effects of the MedDiet are mainly related to its anti-inflammatory and anti-oxidant properties related to the presence of several bioactive compounds [6]. It has also been suggested a role of MedDiet in reducing the risk for the development of neurodegenerative disease [7–9], primarily by decreasing the oxidative stress and inflammation [10] and by modulating the gut microbiota [11] which is linked to the brain through gut-brain axis [12].

One of the most relevant components of MedDiet bioactive compounds are dietary polyphenols. They are secondary metabolites present in fruits, vegetables, cereals, and legumes; they are non-nutritive compounds, but with an important role in the growth of vegetables, also defending the plants from attack by pathogens, predators, and UV radiation [13, 14]. Dietary polyphenols, a complex category with more than 10,000 identified substances, are easily consumed as a part of the diet and of have great scientific interest for their potential health promoting effects. Polyphenols influence also the organoleptic properties of food, including bitterness, astringency, color, flavor, odor, and oxidative stability [13, 14]. Moreover, they can modulate the colonic microbiota [15], affecting the abundance and composition of intestinal microbiota [16]. Fermentation driven by the gut microbiota, in turn, can modify polyphenols into metabolites with enhanced health promoting characteristics. Dietary polyphenols are also widely studied for their role in contrasting aging and age-related diseases.

However, the health promoting effects of dietary polyphenols and their role in disease prevention, are not only dependent on the quantity or to the nature of the ingested polyphenols, but also on the chemical and physical characteristics, which influence their release from the vegetable matrix, their digestive stability in the gastrointestinal (GI) tract, their availability for absorption referred as bioaccessibility [17].

It is noteworthy to consider globe artichokes, capers, and table olives among the traditional functional foods rich in polyphenols and part of MedDiet. All these products are native to the Mediterranean region, where they are traditionally cultivated and consumed as vegetables.

Artichoke (*Cynara scolymus* L.) belongs to the Asteraceae family and has been known since ancient times as both a remedy in medicine and a food that plays an important role in human nutrition. Another vegetable crop typical of the MedDiet is caper (*Capparis spinosa* L.), a perennial bush belonging to the Capperaceae family. Finally, table olives are known to be the most important processed plant food product [18, 19]. Unprocessed olive drupes cannot be readily consumed as they contain compounds responsible for their characteristic bitter taste, such as oleuropein and ligstroside [20]. The most important commercial preparations of table olives are green olives in brine, natural black olives, and olives darkened by oxidation [21].

Many of the traditional products of MedDiet are processed through fermentation. This approach was initially used to preserve, prevent spoilage, extend shelf-life, and improve organoleptic properties of these foods. Over the years, fermentation process effectiveness in improving the functionality of nutrients in food has been demonstrated. Fermented foods contain many components with health benefits and promote the growth of beneficial bacteria favoring digestion, counteracting infection, and even enhancing immune function, thus providing an improvement in health and a reduction of the risk of metabolic disorders and non-communicable diseases [22]. Moreover, microorganisms involved in the food fermentation process have also recently been shown to possess several health benefits [23].

In order to explore novel formulations and recipes to satisfy consumer needs, nutraceuticals-generally considered foods or part of foods capable of providing beneficial health effects - and biotics - including prebiotics, probiotics, postbiotics, and symbiotics - have been widely investigated and applied to various vegetable products [24, 25]. Among biotics, prebiotic substances are defined as “a substrate that is selectively utilized by host microorganisms in the gut, conferring health benefit” [26]. The most recognized and studied prebiotics are fructoligosaccharides (FOS), glucoligosaccharides (GOS), and inulin which can be found in plant-based food matrices. Probiotics have been defined as “live microorganisms that, when administered in adequate amounts, confer a health benefit on the host” [27]. These microorganisms generally include species of *Bifidobacterium* and *Lactobacillus* [28], as well as *Bacillus* species [29]. The interaction between prebiotics and probiotics leads to the creation of a symbiotic, defined as “a mixture comprising live microorganisms and substrate(s) selectively utilized by host microorganisms, that presents a health benefit on the host” [30]. Postbiotics, which do not contain live microorganisms, are substances released by or produced by the microbial metabolism that exert direct or indirect beneficial effects on the host [31]. The specific involvement and demonstration of the therapeutic effects of probiotics and prebiotics in neurodegenerative diseases have been not already widely discussed. However, some experimental data on transgenic mouse models with Alzheimer’s disease have shown changes in the gut microflora, with modified content of metabolites from intestinal bacteria, such as short-chain fatty acids, thus improving cognitive functions [32].

This review focuses on bioactive compounds, specifically polyphenols, that can be found in several functional foods, with particular attention to artichokes, capers and table olives, three traditional Mediterranean products. The use of fermentative approaches to enhance food health benefits is also discussed; and special emphasis is given to the positive effect of plant food polyphenols on aging prevention and reduction. Recent knowledge and applications of polyphenols, their impact on gut microbiota, and their bioavailability are also reported. Finally, an overview of the recently developed applications of probiotics to obtain functional foods with improved nutritional characteristics, quality, and safety traits is also provided.

## Bioactive compounds (polyphenols)

More than 10,000 compounds of dietary polyphenols have been identified in plants, and they are widely studied for their beneficial effect on human health. The chemical structure of polyphenols originates from the common intermediate phenylalanine or from the closely related precursor shikimic acid. They mainly occur in conjugated forms and can have one or more sugar moieties linked to hydroxyl groups, although direct linkage of the sugar (polysaccharide or monosaccharide) to an aromatic carbon has also been demonstrated. Dietary polyphenols can be classified into different groups depending on the number of phenol rings present in their structures and basing on the structural elements that bind these rings to one another. The main classes include phenolic acids, flavonoids, stilbenes and lignans. The presence of polyphenols in plant, tissue and cellular compartments is not uniform. Generally, they are present in both soluble and insoluble forms: the first within the plant cell vacuoles, and the second linked to the cell walls. Some polyphenols, such as the flavonoid quercetin, are quite ubiquitous, being found in nearly all plant products, including fruits, vegetables, cereals, fruit juices, tea, wine, infusions, etc. complex mixtures of polyphenols are present in all foods, while others, such as flavanones and isoflavones, are specific to particular foods. The polyphenol content in plants is affected by several factors, including ripeness, environmental conditions, processing, and storage [33]. Several analytical methods have been developed for the identification and quantification of food polyphenols, leading to an increasing number of recognized compounds. Polyphenols are widely studied for their beneficial actions, primarily considering their antioxidant properties. However, these health benefits are linked not only to the quantity and nature of the polyphenols ingested, but also to their absorption, bioaccessibility and bioavailability.

### Bioaccessibility and bioavailability of polyphenols

Bioaccessibility is defined as the fraction of bioactive compounds released from the matrix in the gastrointestinal tract after the digestion process, and thus potentially available for absorption into the circulatory system [34]. Therefore, the number of bioactive compounds that reach target tissues and exert positive effects on human health represents the bioavailability [35]. Several in vitro methods and procedures have been proposed to evaluate the bioaccessibility of polyphenols, simulating both static and dynamic human gastrointestinal digestion. These methods reproduce the three phases of digestion (oral, gastric, and small intestinal), including a micellization phase for lipophilic compounds [36], and in some cases, are followed by uptake onto Caco-2 cells [37–39]. The in vitro methods simulate the physiological conditions of the human GI tract, respecting the composition of the digestive fluids, pH, and residence time specific to each step [40]. These methods are useful for understanding the mechanism of action of polyphenols; they are highly reproducible, rapid and inexpensive, performed under controlled conditions, and without ethical restrictions. Among the dynamic systems, it is noteworthy to consider the computerized models, such as the TNO Gastro-Intestinal Model (TIM) which includes the gastrointestinal tract model 1 (TIM1) that simulates the upper tract of digestion [41]; this device can be supplemented by the TIM2 system, which mimics the microbial fermentation phases specific to the large intestine and colonic microbiota [42].

Additionally, researchers of CSIRO’s Division of Human and Animal Nutrition (Adelaide, South Australia), developed the NutraScan Artificial Gut, an automated protocol for predicting glycemic index in cereal-based foods [43]. Other systems include SHIME^®^ and M-SHIME^®^ (Prodigest Ghent University, Belgium). These are validated prototypes of dynamic digestion: SHIME^®^ reproduces, under controlled conditions, the physicochemical, enzymatic, and microbial parameters in the GI tract. In addition, the system has also been implemented with a mucosal compartment integrated into the colonic regions (M-SHIME^®^) [44, 45].

These devices are very important to deepen the knowledge of the health promoting effects of polyphenols and to analyze their bioaccessibility, absorption and bioavailability, also considering the modulation of the gut microbiota. Recently, Duque-Soto et al. [46] compared different dynamic gastrointestinal models used to study phenolic compounds from various food matrices and suggested establishing guidelines to improve comparability among studies. These guidelines should include setting minimum and maximum treatment periods for observing effects on colonic microbiota, standardizing volumes and dosages, and creating a recommended protocol for studying phenolic compounds. Additionally, they highlighted the need for more research to understand the impact of dynamic in vitro digestion on the antioxidant capacity of compounds at the gut microbiota level.

**Fig. 1:**
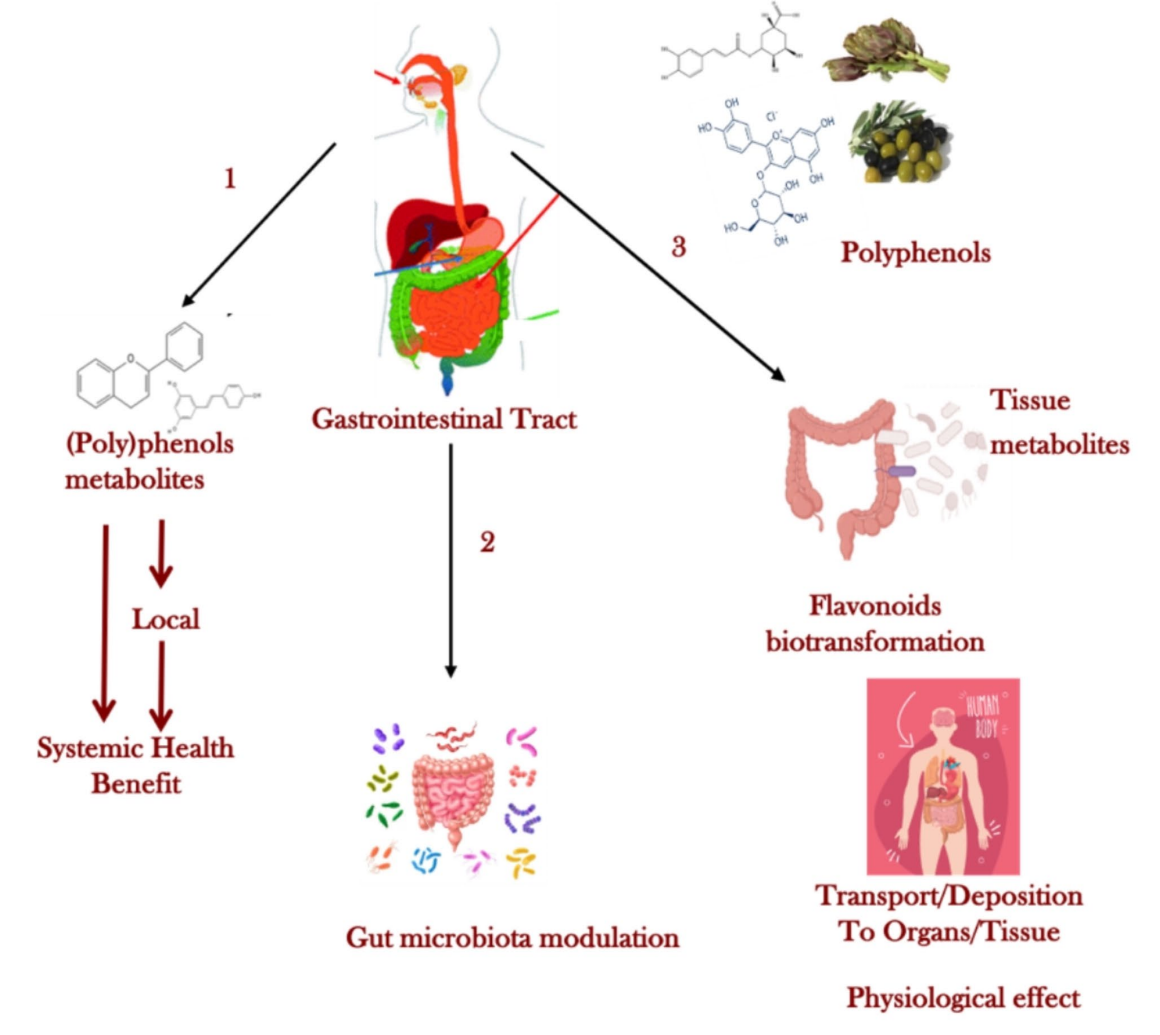
Dietary polyphenols and their fate in the gastrointestinal tract following three different ways. (1) the original compounds with quicky effects favoured by the concentration, (2) influence of the gut microbioma with the modification of polyphenols profiles with possible systemic absorption, (3) tissutal metabolism of the original compound by intestinal epithelial (modified from Rana et al. [14])

Dietary polyphenols are not absorbed in the small intestine, instead they accumulate in the colon where they undergo biochemical transformations (hydrolysis, cleavage, reduction and deglycosylation) resulting in the production of low molecular weight derivatives. that can interact with the gut microbiota and can be easily absorbed, improving both bioavailability and health promoting effects (Fig. 1) [47, 48].

Dietary polyphenols impact gut microbiota composition by promoting the growth of beneficial microbes such as *Lactobacillus* and *Bifidobacterium*, while inhibiting harmful bacteria, i.e. *Clostridium* species. These molecules are metabolized by gut microbiota into bioactive compounds, which contribute to health benefits [49]. The review by Cheng et al. [50] explains polyphenols’ bioactive mechanisms by three ways: regulating gut microbiota composition, influencing the metabolism of microbial metabolites like SCFAs (short-chain fatty acids) and TMAO (trimethylamine N-oxide), and being metabolized by gut microbiota into more bioactive and bioavailable compounds. These mechanisms help explain how polyphenols, despite their limited bioavailability, exert significant health benefits.

The bioavailability of polyphenols is still a crucial factor in evaluating their health promoting effects. It is defined as the fraction of ingested polyphenols that reaches the systemic circulation and the specific compartment or tissues where they can exert their biological action. Bioavailability varies greatly among polyphenols and is not necessarily related to the relative abundance. In general, polyphenol bioavailability is very low compared to others known antioxidants such as vitamins and pro-vitamins (vitamin E, vitamin C, and carotenoids). It is also influenced by the size, presence of sugars, and hydrophobicity of the molecules, which are often excreted in the feces [17, 51, 52].

To determine polyphenols bioavailability, both in vitro (using tissues or human cellular lines) and in vivo (by animal models and human studies) models can be applied. The in vivo studies on animal models provide information that is more comparable to the level of bioavailability in humans [53, 54], though there are limitations primarily due to differences between the animal and human genome and microbiome [55, 56].

## Mediterranean functional foods: artichoke, caper and table olives

### Artichoke

Artichoke is a crop belonging to the Asteraceae family and contributes significantly to the agricultural economy of the Mediterranean area, with an annual production of approximately 770,000 tonnes (over 60% of total global production) from more than 80,000 hectares of cultivated land. Italy is the world’s leading producer (approximately 474,000 tonnes), followed by Spain and France. Artichoke is also cultivated in the Near East (Turkey and Iran), North Africa (Egypt, Morocco, Algeria and Tunisia), South America (Argentina, Chile and Peru) and the United States (particularly in California). Its cultivation is also spreading to China [57, 58].

Artichoke heads are immature composite inflorescences, comprising the edible part of the plant, used worldwide as fresh, frozen, or canned food. They contain approximately 66.3% total carbohydrates, 19.6% protein, 2.0% crude fat, and minerals such as potassium, calcium, sodium, magnesium, phosphorus, iron, copper and manganese [59].

Various parts of the artichoke (stem, leaves, bracts, and flowers) are also characterized by the presence of a number of primary and secondary metabolites, including dietary fibre, polyphenols, flavonoids and terpenoids [59]. The plant also contains sesquiterpene lactones, compounds responsible for 80% of the bitter taste of artichoke leaves [60, 61].

It is also noteworthy that the industrial by-products of the artichoke represent approximately 80% of the biomass and can be used as a raw material for the extraction of food additives such as pectin and antioxidants. Lignocellulosic biomass from artichokes can be used as green fodder for livestock and can also serve as an energy crop [62].

Artichoke fermentation has acquired scientific interest in recent years, leading to numerous studies investigating the chemical, microbiological and nutritional changes that occur during the process. Lactic acid bacteria (LAB) and yeasts interact with the chemical components of the matrix, leading to a series of transformations including sugar degradation, organic acids production, enzyme activation, and the formation of volatile compounds responsible for the characteristic aromas and flavors of the fermented product. The application of selected LAB strains, also with probiotic features, and/or yeast strains for artichoke heads fermentation, is widely studied. These microbial starter strains can control the process and produce artichokes with proper hygienic quality and extended shelf-life [63]. Artichoke fermentation can significantly modify the composition of organic acids, fiber, polyphenols and vitamins as well as it can improve the digestibility and bioaccessibility of the nutrients present in the starting material, thereby enhancing their beneficial effects on health. Depending on the heat treatment applied to the starting material (blanching or slight pasteurization), the fermentation process driven by probiotic LAB strains can affect both the antioxidant activity and the total polyphenols bioaccessibility in the small intestine [64], or it can increase the total polyphenol content and the antioxidant activity (Bleve & Tarantini personal communication).

Artichokes are a rich source of health-promoting compounds such as inulin, fiber, minerals, and polyphenols [65]. These last compounds are well known for their bioactive attributes, including hepato-protective, anticarcinogenic, antioxidative, antibacterial, anti-HIV, bile-expelling, and diuretic properties [66]. Owing to these characteristics, the artichoke can be considered a functional food, according to the definition provided by the European Commission on Functional Food Sciences in Europe (FUFOSE) [67].

The main polyphenols present in artichoke are derivatives of caffeic acid, such as mono and dicaffeoylquinic acids, with chlorogenic acid being the most abundant. Other compounds present in low quantity in artichoke tissues include glycosides of apigenin and luteolin, as well as various cyanidin caffeoyl-glucosides [65, 68]. The artichoke polyphenols stimulate the growth of healthy intestinal bacteria, which may help in preventing some acute and chronic gut disorders. Scientific evidence supported the effect of artichoke extracts in counteracting the Reactive Oxygen Species (ROS) cellular production and protecting the low-density lipoprotein (LDL) from oxidation [69, 70]. Table 1 describes the potential effect of the artichoke polyphenols on human health and the related mechanisms of action.

To confirm the health promoting effects of artichoke polyphenols, it is essential to understand the factors that could influence their release from the vegetable matrix, their degree of absorption, and their fate in the human body. in vitro models have been used to evaluate the bioaccessibility of artichoke polyphenols and their possible modifications in the GI tract. Furthermore, the in vitro GI digestion model has been coupled with the Caco-2 human intestinal cell line, as a tool to predict bioavailability [70]. In the reported paper, the authors demonstrated that the total bioaccessibility of artichoke polyphenol was 55.8%, with chlorogenic acid the most stable and bioaccessible compound (70.0%), followed by 3,5-O- and 1,5-O-di-caffeoylquinic acids (41.3% and 50.3%, respectively), highlighting the susceptibility of these compounds to the conditions of the GI tract. In terms of bioavailability prediction, the authors also identified coumaric acid, caffeic acid and caffeic acid derivatives in the basolateral side, assuming extra and intracellular Caco-2 esterase activities on chlorogenic acid. Only apigenin-7-O- glucoside was found to be bioavailable to an extent of 1.15% after 60 min. The same Caco-2 cells model has been used to evaluate the bioavailability of sesquiterpene lactones and polyphenols from artichoke, thus confirming the nutritional and functional properties of globe artichoke [71]. The beneficial effects of the artichoke were also established using in vivo studies performed by other authors. In particular, Azzini et al. [72] evaluated the absorption, metabolism, and pharmacokinetics of artichoke polyphenols after the oral intake of cooked artichoke heads in humans. The main bioavailable compounds detected by the authors were chlorogenic acid, caffeic acid and ferulic acid, which showed a biphasic profile. After eight hours, the authors observed a significant increase in the total concentration of dihydrocaffeic and dihydroferulic acid; however, no flavonoids were detected in the blood stream [72].

A recent in vivo study on heathy volunteers assuming almost 6 mmol of artichoke polyphenols for 24 h, reported the identification of 76 metabolites in plasma and urine. The authors supposed that a significant amount of artichoke polyphenols reached the colon, where they were metabolized by gut microbiota before absorption. Specifically, chlorogenic acid was metabolized by gut microbiota, producing dihydroferulic acid, 3-(4′-methoxyphenyl) propanoic acid and 3-(3′-hydroxyphenyl) propanoic acid [73].

### Capers

Capers (*Capparis spinosa* L.) are widely distributed across the Mediterranean basin. They are usually not cultivated, as the plants grow spontaneously and exhibit strong resistance to challenging environmental conditions. Their flower buds (capers) and fruits (caper berries) are generally consumed after processing for their flavor, as well as for their digestive and medicinal properties [74, 75].

The unprocessed products of capers (flowers and berries) are characterized by a significant bitter taste due to the presence of polyphenolic compounds and are also subject to rapid deterioration.

In European countries, capers have multiple uses. They are consumed as pickles in salads and sauces, and also as condiments and seasoning spices [76]. Additionally, capers have a long history of ethnomedicinal use worldwide [77].

*C. spinosa* berries contain carbohydrates (5%), dietary fiber (3%), proteins (2%) and lipids (0.9%) and a moderate content of vitamin C (4 mg/100 g fw) [78]. Caper buds and berries are very rich sources of functional bioactive compounds, including phenolic acids, flavonoids, alkaloids, phytosterols, glucosinolates, sugars, vitamins, and organic acids [79].

Caper buds and berries are traditionally fermented by spontaneous LAB in brine before consumption. Although caper processing techniques have recently been applied on an industrial scale, their production remains largely artisanal [80]. Home-made spontaneous fermentation leads to non-reproducible processes, resulting in variations of the sensory properties of the products and potential risks to hygienic quality [81].

Buds or berries are collected during the summer (June and/or July), usually immersed in tap water at ambient temperature, and allowed to ferment for approximately 5 to 7 days at temperatures ranging from 23 °C to 43 °C. After fermentation, capers are placed in brine and distributed for consumption. Alternatively, they can be directly fermented in brine or a mixture of brine and vinegar [77]. Traditionally fermented caper buds and berries are highly appreciated for their unique organoleptic properties, and are often consumed as an appetizer or used as ingredients in the preparation of traditional foods. They also have desirable new flavors and specific textures.

In commercial fermented caper preparations, high levels of phenolic compounds, such as rutin, quercetin 3-rutinoside, kaempferol 3-rutinoside; and kaempferol 3-rhamnosyl-rutinoside have been detected [82]. Rutin, also known as vitamin P, is a plant phenolic compound recognized for its antioxidant, anti-inflammatory and anticarcinogenic effect, as well as for its ability to reduce blood vessels fragility [83]. Tocopherols, belonging to the vitamin group, and carotenoids are also important antioxidants and represent the most abundant group of lipid antioxidants observed in fermented caper preparations. Among volatile compounds, aldehydes and esters are the most abundant classes, with methyl-isothiocyanate being the major one, followed by benzyl-isothiocyanate [84].

Furthermore, fermentation can facilitate the release of phenolic compounds from the plant matrix, thereby increasing the antioxidative activity of the fermented product. The use of starter cultures has been proposed as an appropriate approach for (i) controlling and optimizing the fermentation process, (ii) mitigating problems related to variations in organoleptic and hygienic quality, (iii) improving the nutritional traits of the final product [85]. In fact, process control and standardization are needed to enhance fermentation and produce high-quality end products. The use of a selected *Lactiplantibacillus plantarum* strain on caper berries has enabled the production of a consistently homogeneous product with desirable and controlled properties, while reducing the risk of spoilage or stuck fermentation [80].

Other studies have shown that fermentation can significantly change the polyphenol profile of capers and produce high levels of quercetin derived from rutin hydrolysis [86].

The importance of controlling and standardizing the fermentation was highlighted by the discordant data obtained in the study reported by Sonmezdag et al. [87] where the process affected the aroma compounds, total phenolic concentration, and antioxidant activity of caper buds.

In the case of caper berries, traditional spontaneous fermentation was driven by LAB, with *L. plantarum* as the predominant species, along with others belonging to *Bacillus*,* Enterococcus*,* Weissella*,* Pediococcus* genera, producing different effects on nutrient compounds. During fermentation, glucosinolates were fully degraded, quercetin glycosides partially hydrolysed, and epicatechin content reduced, while the antioxidant activity was generally maintained or slightly increased [88]. Errachidi et al. [89] also demonstrated that caper berries processed following the traditional fermentation method were characterized by a high antioxidant capacity and high content of crude protein, phenolic compounds, and flavonoids.

Ozcan and Uslu [90] reported that the spontaneous fermentation process of caper buds carried out in the presence of additives (salt, glucose, honey, sugar) resulted in a decrease of carotenoid content and an increase in some phenolics (resveratrol, quercetin and kaempferol). Similarly, the inoculation of LAB starter culture and the use of modified brines (with lactic acid addition) enhanced the dry matter, protein, and mineral content of fermented caper buds [91].

The dry-salting fermentation methods of caper buds produced significant levels of antioxidant activity, with a notable increase in quercetin, kaempferol, and isorhamnetin compounds compared to untreated fresh samples [75].

Recent evidence suggests that *C. spinosa* buds can be considered as functional food. In fact, the development of new treatment procedures and the use of specific LAB strains offer the opportunity to stabilize the final products in short time periods, while also ensuring an increase in important nutritional traits (total phenolic content and total antioxidant activity) and aroma and flavor components (Bleve & Tarantini personal communication).

As described, the flower buds of *Capparis spinosa* L. (capers) have attracted the attention of various authors, who have thoroughly analysed their bioactive components, particularly polyphenols, for their health benefits, including antioxidant, anticarcinogenic, antimicrobial, and antimutagenic properties [92, 93]. The polyphenol composition of capers depends on genotype, growing stage, and geographic area. Caper flower buds and berries contain high amounts of rutin and quercetin. These compounds are of great importance for their ability to scavenge free radicals, thus contributing to their beneficial biological properties [94].

Several authors have reported that in five caper cultivars typical of Pantelleria area, the total polyphenols content (TPC) ranged from 596.92 mg gallic acid equivalent (GAE)/100 g dry matter (DM) to 843.92 mg GAE/100 g DM. In addition, the total flavonoids amount (TFC) ranged from 20.65 ± 0.01 to 90.31 ± 0.01 mg resveratrol equivalent (RE)/100 g DM [95]. Other authors [96] reported different results on caper cultivars typical of Turkey region, with a TPC of 465 mg GAE/100 g and TFC of 55.3 ± 4.2 mg catechin equivalent (CE)/100 g. Furthermore, Wojdyło et al. [97] found that in other caper cultivars from Spain, the TPC ranged from 10,720 to 3,256 mg/100 g DM. These data demonstrated that the region of production and cultivar can influence the polyphenols content. From qualitative characteristics, the chromatographic profile of raw caper buds showed the presence of phenolic acids and flavonoids. In particular, p-coumaric, ferulic, 4-hydroxybenzoic, vanillic, syringic, protocatechuic, and sinapic acids, have been identified among the phenolic acids. Regarding flavonoids, rutin, kaempferol-3-O-rutinoside, narcissoside, hyperoside, kaempferol, quercetin, taxifolin, eriodictyol, and hesperidin, together with derivatives of kaempferol and quercetin, have been identified [95, 96]. As reported for the other vegetable matrices analysed in this review, the identified polyphenols in capers can confer health benefits, as demonstrated by several studies (Table 1).

The bioaccessibility of polyphenols is a crucial point to evaluate their human health promoting influence. To date, there are limited scientific evidences on the caper polyphenols bioaccessibility, and most of them are addressed to evaluate the TPC bioaccessibility [98, 99]. Recently, Berkel Kaşıkçı et al. [96] determined the caper polyphenols bioaccessibility by applying a static in vitro gastrointestinal digestion on two different samples: the raw and the pickled capers, with a resulting bioaccessibility of 77.8% and 72.9%, respectively. Furthermore, among the identified flavonoids, rutin and kaempferol-3-O-rutinoside were found as the main bioaccessible ones, with an exert of 45.3% for rutin in the raw caper, and between the 95 and 97.4% for the kaempferol-3-O-rutinoside in both raw and pickled samples. Hyperoside was quite stable in the raw caper with a bioaccessibility of more than 100% (107.8%), while the bioaccessibility of narcissoside in the raw caper sample was 67.7%. In the pickled capers, all detected phenolic acids were bioaccessible: 4-hydrobenzoic acid and vanillic acid showed levels of 184.7% and 118.2%, respectively, followed by ferulic acid (65.2%) and narcissoside (47.5%). This result could be attributed to the release of polyphenols from the food matrix to the brine medium during fermentation. Nevertheless, the overall results suggest that capers are a good source of bioaccessible polyphenols with potential heath promoting effects. However, further studies are needed to explore the bioavailability of these polyphenols and their potential impact on microbiome modulation.

### Table olives

In the food industry, table olives are one of the most important fermented plant products [18]. According to estimates by the International Olive Council, Spain, Greece, and Italy account for approximately 30% of the world’s annual production [19].

Unprocessed olive drupes are not readily consumable due to the presence compounds responsible for their characteristic bitter taste, such as oleuropein and ligstroside [20]. On the international market, key commercial preparations of table olives include green olives treated in brine, natural black olives, and olives darkened through oxidation [21].

At the end of processing, table olives can be preserved as bulk preparations through careful management of several parameters, including pH (typically < 4.3), salt concentration (at least 6% w/v sodium chloride or more), and acid levels (i.e., lactic acid 0.3% w/v) [21].

In recent years, table olives have garnered increasing interest due to their purported health benefits. These lasts appear to be intrinsically linked to olives high content of monounsaturated fatty acids, as well as their antioxidant capacity, derived from minor compounds such as tocopherols and phenols [100]. In the traditional natural black olives production, the fruits are placed directly in brine with a salt concentration of 6–10% w/v. Then, spontaneous fermentation, driven by epiphytic microflora of yeasts and LAB, proceeds for several months [101, 102].

In this context, the use of starter cultures, including the use of selected LAB strains [103, 104] has been proposed to control, standardize and improve the fermentation process. However, the complex selection process and their subsequent industrial validation have limited the use of starter cultures [105].

LAB metabolism is fundamental in the production of table olives for human consumption, as it promotes the debittering of olives, prevents the growth of spoilage and pathogenic microorganisms [106, 107], and enhances the flavor and texture profile in the final products [108]. Meanwhile, yeasts play significant roles in producing volatile compounds and metabolites, thus improving the organoleptic characteristics of the final product [109]. Moreover, yeast can contribute to releasing nutrient compounds that promote LAB growth [110, 111] and counteract the development of undesired microorganisms [112].

The use of starter cultures for table olive fermentation, whether single or composite preparations, is becoming increasingly attractive for the food industry, as it helps reduce costs, decrease fermentation time, minimize spoilage risks, and improve the safety and sensory characteristics of the products [113, 114].

The natural black olives fermentation method has been found to result in a higher content of total phenols compared to other processing methods. The fate of polyphenols can be influenced by the microbial fermentation and by the thermal processing methods applied. Heat treatments, such as pasteurization or hot bottling, are generally used to minimize the risk of food-borne diseases, reduce vegetative organisms, and extend the product’s shelf-life [115].

In this regard, two effects may occur: the mobilization of bound phenolics from the plant cell walls, and the oxidation or degradation of phenolics species more susceptible to heat exposure [116]. However, the use of selected yeasts and bacteria for fermentation is promising for producing a nutritionally improved final product with increased levels of keyphenolic compounds and related antioxidant activity.

Table olives are considered a source of bioactive compounds such as polyphenols with associated health promoting activities (Table 1). The main classes of polyphenols identified in table olives are phenolic acids, phenolic alcohols, phenylpropanoids, flavonoids, and secoiridoids. The most abundant phenolic compounds are hydroxytyrosol and tyrosol, the desterification products of oleuropein and ligstroside, followed by the phenylpropanoids verbascoside and isoverbascoside, and different flavonol glycosides such as rutin, luteolin-7-glucoside, and apigenin-7-glucoside [40]. Recent studies have demonstrated that naturally fermented table olives can be an excellent source of bioactive compounds, even compared to olive oil, for which a nutritional EU claim (No 433/2012 of 23 May 2012) has been established [117]. This claim confers to olive oil polyphenols the ability to protect the blood lipids from oxidative stress. Specifically, to meet the claim, a daily intake of 20 g of olive oil, containing at least 5 mg of hydroxytyrosol and its derivatives (e.g., oleuropein complex and tyrosol) is required. This effect allows producers to indicate the EU specific health claim on the label [118]. The study of D’Antuono et al. [117] showed that an intake of 20 g of naturally fermented table olives provided an introduction of hydroxytyrosol and its derivatives, ranging from 7 to 40 mg, almost eight times higher than in 20 g of olive oil. These evidences indicate that naturally fermented table olives may have a comparable health promoting effect to olive oil. This assessment could be the starting point for considering naturally fermented table olives as functional foods characteristics of MedDiet, and enhancing consumer awareness of this food commodity. Linked to this point, evaluating the bioaccessibility and bioavailability of table olives polyphenols is crucial for understanding their health promoting effect. Some applied in vitro methods, which include simulated GI digestion coupled with the Caco-2 cell model, are utilized to assess these parameters. In particular, the results obtained by D’Antuono et al. [40] on naturally fermented table olives cv *Bella di Cerignola* highlighted that the bioaccessibility of polyphenols ranged from 7% of luteolin to 100% of tyrosol. These results underscored the high susceptibility of flavonoids to digestive conditions. Regarding bioavailability, polyphenol accumulation in Caco-2/TC7 cells was rapid (60 min) but occurred at low efficiency (0.89%), with hydroxytyrosol and tyrosol being the most bioavailable compounds, followed by verbascoside and luteolin.

The same authors compared the effects of fermentation by autochthonous microbial starters on the phenolics composition of three Apulian table olives, *Bella di Cerignola*, *Termite di Bitetto* and *Cellina di Nardò*, highlighting the influence of the cultivar. The authors also assessed the polyphenols’ in vitro bioaccessibility (> 60%) noting a modification in their profile after GI digestion, with the presence of newly released phenolic compounds, likely due to both the environment conditions and the microbial influence [117].

**Table 1:** Main phenolic compounds present in artichoke, caper and table olives their potential effect on human health and related mechanisms of action

	Polyphenols	Activity	Mechanism of action	Reference
Artichoke	*Mono and dicaffeoylquinic acids* Chlorogenic acid 1,3 dicaffeoylquinic acid (Cynarin) 1,5 dicaffeoylquinic acid, 3,5 dicaffeoylquinic acid *Flavonoids* Apigenin Luteolin *Anthocyanins* Cyanidin Peonidin Delfinidin	Hepatoprotective effect	Hepatocytes protection from oxidative stress, detoxification and improvement of liver function	[119]
		Antioxidant, cardioprotective and neuroprotective effects	Free radical scavenger, inhibition of lipid peroxidation, Inibition of acetylcholinesterase (AChE); inhibition of TMAO formation	[120–122]
		Antiaging activity	Improvement of the integrity and functionality of endothelial cells	[123]
		Choleretic activity with hypocholesterolemic effect	Increased bile flow with elimination of cholesterol Inhibition of hepatic Chl synthesis by CoA reductase modulation of the HMG enzyme	[124–126]
		Anti-atherosclerotic effect	Inhibition of LDL oxidation, promotion of the synthesis of the antithrombotic and antiatherosclerotic enzyme eNOS	[127, 128]
Caper	*Phenolic acids* p-Coumaric acid Sinapic acid 4-hydroxybenzoic acid Vanillic acid Syringic acid Ferulic acid Protocatechuic acid *Flavonoids* Rutin Kaempferol-3-O-rutinoside Quercetin Narcissoside Hyperoside Kaempferol Taxifolin Eriodictyol Hesperidin	Antioxidant effect	Protection against free radical damage	[75, 94, 129]
		Antidiabetic activity	Reduction of fast blood glucose levels and increase of insuline secretion and glucose absorption from the small intestine	[130, 131]
		Hepatoprotective and Neuroprotective effects	Increasing levels of detoxification enzymes and decrease of enzyme of damage (ALT, AST, AKP, γ-GT, LDH); Regulation of inflammation-involved genes in Alzheimer’s	[132, 133]
		Anti-cancer effect	Controlling the proliferation, differentiation and apoptosis of various tumour cells; Suppression of NF-κB transcription factor	[92, 134]
		Antimicrobial effect	Inhibition of the growth of different bacterial patogens	[135, 136]
Table Olives	*Secoiridoids* Oleuropein *Phenolic alcohols* Hydroxytyrosol Tyrosol *Phenylpropanoids* Verbascoside *Phenolic acids* Caffeic acid Vanillic acid *Flavonoids* Luteolin Apigenin *Anthocyanins* Cyanidin	Antioxidant effect	Prevention of oxidative damage, chelation of metal ions that catalyze the formation of radicals, 5-lipoxygenase inhibition	[137, 138]
		Anti-atherosclerotic effect	Scavenging of H_2_O_2_, that leads to platelet aggregation; oxidation protection of biological membranes	[139, 140]
		Hypoglycemic, antihypertensive effect	Increase of peripheral glucose absorption, influence on insulin release	[141, 142]
		Anticancer effect	Inhibition of tumor cell proliferation and migration	[143]

## In vitro and in vivo assessment of bioactive compounds efficacy in antiaging disease prevention

The mechanisms of aging are likely driven by free radical/oxidative stress, as reported by different authors [144, 145]. Although oxidative conditions are normally present, the cellular damages become more significant during aging due to the decreased ability of the cellular system to counteract damage and to the decline of the tissue repair processes. The authors also highlighted that a diet rich in antioxidants, such as polyphenols from plant-derived functional foods, can mitigate the negative effects of aging [145] due to their well-known anti-inflammatory and antioxidant activities [14], thereby promoting healthy aging and preventing neurological disability. Some polyphenols can cross the blood-brain barrier, reach the brain, and play an important role in reducing neurodegeneration [146].

Among the polyphenols, hydroxytyrosol (HT) has received particular attention for its positive health impact due to its bioavailability and various biological properties, including anti-inflammatory, antimicrobial, antiviral, antifungal, cardioprotective, neuroprotective, antitumoral, and chemo-modulating effects, as well as its low toxicity [147]. In the context of age-related risks such as dementia, Alzheimer’s disease, and Parkinson’s disease, several studies have demonstrated the ability of HT to easily cross the blood-brain barrier [148]. Some in vivo studies on adult and aged wild-type mice have demonstrated that HT contributes in neurogenesis. The results revealed that HT treatment activated hippocampal neurogenesis in the dentate gyrus of all treated mice, increased the survival of new neurons, decreased apoptosis, and improved the proliferation of stem and progenitor cells, without causing any proliferative effects in the dentate gyrus of adult [149]. Recently, the same authors analyzed the effects of HT and/or oleuropein (OLE) in delaying and preventing several age-related diseases, such as neurodegenerative and neurological disorders, skeletal muscle and bone deterioration, and altered metabolism. They concluded that, in experiments performed on model organisms, both compounds can ameliorate lifespan by counteracting cellular senescence and modulating the gut microbiota [150].

Furthermore, polyphenols from artichoke heads may also exert antiaging and neuroprotective effects, potentially serving as dietary supplements as adjuncts to conventional therapies. Abd El-Aziz et al. [151], demonstrated that artichoke polyphenols exerted an inhibitory effect against acetylcholinesterase (AChE), an enzyme responsible for the hydrolysis of acetylcholine, a physiological reaction necessary for the cessation of nerve impulse transmission. In particular, in the Alzheimer disease, one of the most common causes of dementia in older adults, cholinergic neurons degenerate in specific brain areas related to intellectual functions, memory, and consciousness. Drugs as galantamine, donepezil, rivastigmine, which are used as AChE inhibitors, have been shown to treat this condition. Recent studies suggested that artichoke or its polyphenols could be used as adjuvants of drug therapies for patients with neurodegenerative disorders [122]. Other studies have demonstrated the ability of artichoke polyphenols to improve the integrity and functionality of endothelial cells, highlighting their antiaging capacity [123]. Artichoke polyphenols also have a significant effect on inhibiting LDL oxidation, promoting the synthesis of the antithrombotic and anti-atherosclerotic enzyme eNOS [127, 128].

Similarly, polyphenols from caper flowers have been shown to reduce inflammation, particularly in relation to aging protection. Some studies evaluated the antioxidant power of caper polyphenols using different in vitro methods. Additionally, the ability of caper flowers to inhibit acetylcholinesterase (AChE) and butyrylcholinesterase (BuChE) has also been studied [97]. The authors demonstrated that the inhibition of these two enzymes’ activities was directly related to the prevention of the development and progression of Alzheimer’s disease and depended on the cultivar and its growth stage. The results reported by the authors also suggest that the extracts capable of inhibiting BuChE, can also prevent the accumulation of the β-amyloid protein, which is responsible for the progression of Alzheimer’s, by helping to prevent the spread of β-amyloid plaques [152].

## Probiotic enriched vegetable products

Among functional foods, probiotic enriched products constitute a significant portion. Nowadays, the consumption of probiotic foods is increasing due to the demand for a healthy and safe diet. The global probiotics market has grown considerably over the last few decades, from approximately $ 16 billion in 2008 [153] to $ 34.1 billion in 2020, and is expected to reach $ 73.9 billion by 2030 [154]. This trend suggests integrating probiotics into the MedDiet. Currently, most commercially available probiotic foods are milk based, but there is an increasing demand for lactose- and cholesterol-free options. Plant-based food matrices, which can serve as carriers of probiotic cells to the human gut, have recently been explored as lactose-free alternatives [155–158]. The minimum daily dose for probiotics is 10^9^ CFU for portion, corresponding to 10^7^ CFU/g or CFU/mL [27]. To achieve this threshold in the final food product, probiotic strains must overcome several challenges, from food process to GI transit. The ability of a probiotic strain to survive food processing and harsh gastro-intestinal conditions depends on strain-specific characteristics and several factors related to the food matrix used as a carrier. The food constituents and structure can significantly protect cells and contribute to the implantation of live probiotic cells on food surfaces. The processes developed thus far for enriching food with live probiotic cells vary depending on the type of food being produced [159]. Fermentation is the oldest and most widely used biotechnological approach, adaptable and optimizable for specific food. Examples of probiotic fermented foods include those milk-based [157] but mild fermentation technologies have also been developed for vegetables [160, 161]. Other technologies include the application of microencapsulated and nano-encapsulated probiotic cells during the food process [162, 163], brining steps [164], the incorporation of probiotic cells into edible coating [165], vacuum impregnation [166–168], and emerging technologies [169].

The methods for incorporating probiotic cells generally involve the simple addition of the culture to the juice, with or without subsequent fermentation. For solid foods, the technology may involve applying an edible coating, fermentation, and microencapsulation (Table 2). A few studies have been focused on minimally processed probiotic vegetable foods; in particular, the suitability of table olives, cabbage and artichokes to support the growth of a probiotic strain (*Lacticaseibacillus paracasei* IMPC2.1) and facilitate its transport and survival through the GI tract has been demonstrated [160, 161, 170]. The procedure for producing these vegetable products includes a mild fermentation step without pasteurization, resulting in typical Mediterranean diet products enriched with probiotic cells that can be consumed daily.

### Probiotic bacteria can strengthen the health-promoting features of functional foods

An overview of the main outcomes of the beneficial effects of probiotic vegan products, both in vitro and in vivo, has been reported by Pimentel et al. [171]. Most studies to date have focused on fruit or vegetable juices and beverages, with the main health effects being the impact on intestinal barrier integrity, *Helicobacter pylori*-associated diseases and anticarcinogenic properties, cholesterol lowering capacity, improved immunomodulatory activity, modulation of the gut microbiota, and alleviation of perennial allergic rhinitis. However, only a few studies have been conducted on human subjects consuming probiotic foods.

Among in vitro studies, Sireswar et al. [172] investigated on the interaction between probiotic and fruit phenolics and the mitigation of the risk and morbidity associated with some chronic diseases. The authors demonstrated a restorative potential on the intestinal barrier function and mucosal damage in zebrafish (*Danio rerio*), after administration of a malt-supplemented sea buckthorn beverage enriched with *Lactobacillus rhamnosus* GG.

Siripun et al. [173] investigated the effect of consuming vegetable and fruit juice composed of a mixture of fruit (apple) and vegetables (green lettuce, celery, cherry tomato, onion) with (probiotic group) and without probiotic *Lactobacillus paracasei* (placebo group), on the body weight, body mass index, waist circumference, lipid profile, lipid peroxidation, oxidative stress enzymes, and bile acid level in dyslipidemic patients. Results indicated a significant effect of the probiotic vegetable fruit juice, administered at the dosage of 2 × 10^9^ CFU/day, on total cholesterol, low-density lipoprotein cholesterol, triglyceride (TG), and TG/high-density lipoprotein cholesterol (HDL-C) ratio. The HDL-C concentration in the probiotic group was higher than in the placebo group. The probiotic group showed significantly decreased malondialdehyde levels, increased oxidative stress enzymes (catalase, and glutathione peroxidase) in plasma, and increased bile acid levels in the feces.

Recently, the combination of MedDiet with probiotic supplementation has been considered for slowing mild cognitive impairment (MCI) [174]. The study mainly described the methods, participant selection and recruitment, adherence strategies, and baseline characteristics of a randomized, latin-square crossover, double-blind, controlled dietary intervention trial performed in MCI patients. However, the study presented some limitations inherent to the long-term intervention studies, which did not allow for significant results in clinical parameters. Nevertheless, it could represent a starting point for evaluating the association between the MedDiet and probiotics in specific subjects to study the relationship between the brain and gut.

In vivo human trials demonstrated the efficacy of ready-to-eat artichokes in ameliorating the constipation symptoms, modulating intestinal microbiota, and altering metabolite pattern in humans [175, 176]. A double-blind, controlled, crossover study conducted by administering probiotic ready-to-eat artichokes to human subjects suffering from constipation by Riezzo et al. [175], demonstrated the functional benefits of the probiotic enriched vegetable food in reducing constipation symptoms. This study is the only in vivo study employing a plant-based probiotic food included in the systematic review and meta-analysis by Dimidi et al. [177], which aimed to investigate the effect of probiotics on functional constipation in adults. No adverse events were reported in either the probiotic or the placebo group, and > 95% compliance was achieved with the probiotic- containing artichokes, confirming that the probiotic addition did not affect the sensory quality of the product. Simultaneously, the functional efficacy of probiotic ready-to-eat artichokes on the fecal microorganisms and related metabolism has been evaluated in Valerio et al. [176]. The study suggested a significant role of the probiotic strain *Lacticaseibacillus paracasei* IMPC2.1 in positively modulating the microbial profile by antagonizing *Escherichia coli* and *Clostridium* spp. and increasing the genetic diversity of the LAB population.

**Table 2:** Research studies aimed to develop probiotic enriched fruit products and main results

Vegetable matrix	Probiotic inclusion method	Probiotic strain	Main Results	References
Artichoke	Inoculation of brine - surface adhesion	*L. paracasei* IMPC2.1 (LMG-P22043)	The probiotic strain: - survived on the matrix with a load ≥ 7 log CFU/g - survived simulated gastro-intestinal digestion - transiently colonized the gut of 17 ⁄ 20 subjects - antagonized *E. coli* and *Clostridium* spp. - increased the genetic diversity of lactic population - improved symptoms of constipation	[161]
Dried Pumpkin	Dry infusion process for iron and edible coating for probiotic incorporation	*L. casei*	The probiotic concentration remained > 10^7^ CFU/g for 14 days and the viability was affected by the mineral incorporation. The iron bioaccessibility was improved by the presence of *L. casei.*	[178]
Table olives	Inoculation of brine - surface adhesion	*L. paracasei* IMPC2.1	The probiotic strain: - colonized the olive surface dominating the natural LAB population - survived on the matrix with a load ≥ 7 log CFU/g - survived simulated gastro-intestinal digestion. - A final low-salt-probiotic product was obtained	[160]
	Inoculation of brine - surface adhesion -	*L. pentosus* B281, *L. plantarum* B282	The strains were ≥ 6 log CFU/g on olive drupes. - *L. pentosus* B281 and *L. plantarum* B282 showed a high survival rate on the matrix	[179]
	Inoculation of brine - surface adhesion	*L. pentosus* TOMC-LAB2	A favorable effect on fermentation and strain predominance was observed by: - an immediate post-brining inoculation - the use of a re-inoculation - an early processing in the season	[180]
Table olive paste	Incorporation of microencapsulated probiotic cells into the olive paste	*L. plantarum* 33	Microencapsulation with sodium alginate and starch - Encapsulation conferred additional protection to *L. plantarum* 33 (about 7 log CFU/g), when exposed to simulated gastro-intestinal conditions. - Microencapsulation did not adversely affect adhesion capacity to intestinal epithelium. - Microcapsules incorporated in olive paste did not affect physicochemical and sensory properties	[181]
Cabbage	Inoculation of brine - surface adhesion -	*L. paracasei* IMPC2.1	The final product contained about 8 log CFU/g of the strain. - Blanching before fermentation preserved glucosinolates. - The acidification performed by the probiotic ensured a microbiological stabilization of the product	[170]
Sauerkraut (Cabbage)	Inoculation of brine - surface adhesion -	*L. plantarum* L4, *Leuc. mesenteroides* LMG 7954	Viable probiotic cells count in final product was ≥ 6 log CFU/g of product. - The strains were used as starter cultures for fermentation allowing a NaCl reduction from 4.0–2.5% (w/v)	[182]
Fresh-cut carrot	Sodium alginate coating	*L. acidophilus* La-14	Counts of the probiotic was ≥ 7 log CFU/g. - The coating contributed to the quality of the minimally processed carrots by conserving their moisture and minimizing acidity variation and color changes during storage	[183]
Fresh or dried beetroot	Spraying - surface adhesion	*L. plantarum* MIUG BL3	Dried chips: strain load was greater than 7 log CFU/g. - Fresh cubes: strain load was greater than 8 log CFU/g	[184]

*B.: Bifidobacterium; E.: Escherichia; L.: Lactobacillus; Leuc.: Leuconostoc; Ls.: Listeria; S.: Saccharomyces. Lc: Lactococcus; P.: Pediococcus*

## Conclusions & future perspective

In conclusion, this review highlights the influence of the Mediterranean Diet (MedDiet) on improving human health and/or maintaining well-being, with particular emphasis on reducing the effects of aging. Vegetable foods typical of MedDiet, such as globe artichokes, capers and table olives containing bioactive compounds, mainly polyphenols, were considered for their potential influence on aging reduction and prevention, also discussing their effect on intestinal microbiota. Indeed, micronutrients such as polyphenols can modulate the microbiota composition and balance, exerting inhibitory and/or prebiotic-like effects. Additionally, microbial groups can transform polyphenols into more absorbable forms, leading to the formation of new active metabolites. Understanding the interactions between bioactive food compounds and specific intestinal microorganisms could firstly contribute to a better understanding of both positive and negative in vivo interactions. Then the microbiota-polyphenols relationship could enhance their bioavailability, ultimately paving the way for personalized nutrition (optimal dose and specific microorganisms). Further research is necessary to explore the bidirectional role and the behavior of polyphenols with respect to the microbial population. This last can provide new insight into the development of treatments for central nervous system disorders. Food products belonging to the MedDiet can be “functionalized” through biotechnological approach such as fermentation or fortification with live probiotic cells. The fermentation process of the vegetables examined in the reported “case-studies” significantly modifies their chemical and biological composition. The fermentation process resulted in enhancing the recovery of bioactive molecules, improving their bioaccessibility and bioavailability, and thereby increasing their beneficial health effects. Moreover, fermentation is useful to produce consistently homogeneous product with desirable and controlled properties, also stabilizing the final products in short time periods. This process reduces the risk of spoilage or stuck fermentations while simultaneously enhancing important nutritional traits and aroma and flavor components. The results reported for globe artichoke, table olives, and capers indicate that the mild fermentation approaches without pasteurization preserves the nutritional trait of Mediterranean foods enriched with probiotic cells, making them suitable for daily consumption. Given the high diversity of food matrices and their structural and composition complexity, incorporating probiotic remains a significant technological issues for researchers and food companies. The main challenges to be addressed for the development of novel probiotic foods include: (i) selecting the most suitable microbial strain, (ii) developing a sustainable and mild food process to incorporate live probiotic cells into or onto the food matrix, and (iii) assessing their health efficacy through human trials. Therefore, further research should focus on exploring various food matrices that could serve as probiotic carriers and on developing new technologies for probiotic incorporation to expand the range of functional foods and “functional diet”. To date, several in vivo studies have demonstrated the influence of the MedDiet on human well-being, even when specific food items are involved. The biggest challenge lies in providing in vivo evidence of the effects of a complete dietetic pattern on healthy aging and understanding the underlying mechanisms of action. Consequently, further investigations should be performed to evaluate the impact of both functional and traditional MedDiet on human health.

## Data Availability

No datasets were generated or analysed during the current study.

## References

[1] Schwingshackl L, Morze J, Hoffmann G (2020) Mediterranean diet and health status: active ingredients and pharmacological mechanisms. Br J Pharmacol 177:1241–1257. 10.1111/bph.14778 31243760 10.1111/bph.14778 PMC7056467

[2] Finicelli M, Squillaro T, Di Cristo F, Di Salle A, Melone MAB, Galderisi U, Peluso G (2019) Metabolic syndrome, Mediterranean diet, and polyphenols: evidence and perspectives. J Cell Physiol 234:5807–5826. 10.1002/jcp.27506 30317573 10.1002/jcp.27506

[3] Finicelli M, Di Salle A, Galderisi U, Peluso G (2022) The Mediterranean Diet: an update of the clinical trials. Nutrients 14:2956. 10.3390/nu14142956 35889911 10.3390/nu14142956 PMC9317652

[4] Davis C, Bryan J, Hodgson J, Murphy K (2015) Definition of the Mediterranean diet: a literature review. Nutrients 7:9139–9153. 10.3390/nu7115459 26556369 10.3390/nu7115459 PMC4663587

[5] Diplock AT, Action EC (1999) Scientific concepts of functional foods in Europe: consensus document. Brit J Nutr 81:S1–S27. 10.1079/BJN19990002 10999022

[6] Ortega RM (2006) Importance of functional foods in the Mediterranean diet. Pub Health Nut 9:1136–1140. 10.1017/S1368980007668530 10.1017/S1368980007668530 17378953

[7] Singh B, Parsaik A, Mielke M et al (2014) Association of Mediterranean diet with mild cognitive impairment and Alzheimer’s disease: a systematic review and meta-analysis. J Alzheimers Dis 39:271–282. 10.3233/JAD-130830 24164735 10.3233/JAD-130830 PMC3946820

[8] Santoro A, Pini E, Scurti M, Palmas G et al (2014) Combating inflammaging through a Mediterranean whole diet approach: the NU-AGE project’s conceptual framework and design. Mech Ageing Dev 136–137:3–13. 10.1016/j.freeradbiomed.201308.109 24342354 10.1016/j.mad.2013.12.001

[9] Coelho-Junior H, Trichopoulou A, Panza F (2021) Cross-sectional and longitudinal associations between adherence to Mediterranean diet with physical performance and cognitive function in older adults: a systematic review and meta-analysis. Ageing Res Rev 70:101395. 10.1016/j.arr.2021.101395 34153553 10.1016/j.arr.2021.101395

[10] Iadecola C, Duering M, Hachinski V et al (2019) Vascular cognitive impairment and dementia: JACC scientific expert panel. J Am Coll Cardiol 73:3326–3344. 10.1016/j.jacc.2019.04.034 31248555 10.1016/j.jacc.2019.04.034 PMC6719789

[11] Rejeski J, Wilson F, Nagpal R, Yadav H, Weinberg R (2021) The impact of a mediterranean diet on the gut microbiome in healthy human subjects: a pilot study. Digestion 103:133–140. 10.1159/000519445 34749376 10.1159/000519445 PMC8916822

[12] Cryan J, O’Riordan K, Cowan C et al (2019) The microbiota-gut-brain axis. Physiol Rev 99:1877–2013. 10.1152/physrev.00018.2018 31460832 10.1152/physrev.00018.2018

[13] Bié J, Sepodes B, Fernandes P, Ribeiro MH (2023) Polyphenols in health and disease: gut microbiota, bioaccessibility, and bioavailability. Compounds 3:40–72. 10.3390/compounds3010005

[14] Rana A, Samtiya M, Dhewa T, Mishra V, Aluko RE (2022) Health benefits of polyphenols: a concise review. J Food Biochem 46:e14264. 10.1111/jfbc.14264 35694805 10.1111/jfbc.14264

[15] Lippolis T, Cofano M, Caponio GR, De Nunzio V, Notarnicola M (2023) Bioaccessibility and bioavailability of diet polyphenols and their modulation of gut microbiota. Int J Mol Sci 24:3813. 10.3390/ijms24043813 36835225 10.3390/ijms24043813 PMC9961503

[16] Bode LM, Bunzel D, Huch M et al (2013) In vivo and in vitro metabolism of *trans*-resveratrol by human gut microbiota. Am J Clin Nutr 97:295–309. 10.3945/ajcn.112.049379 23283496 10.3945/ajcn.112.049379

[17] Manach C, Williamson G, Morand C, Scalbert A, Rémésy C (2005) Bioavailability and bioefficacy of polyphenols in humans. I. Review of 97 bioavailability studies. Am J Clin Nutr 81:230S–42S. 10.1093/ajcn/81.1.230S 15640486 10.1093/ajcn/81.1.230S

[18] Caponio F, Difonzo G, Calasso M, Cosmai L, De Angelis M (2019) Effects of olive leaf extract addition on fermentative and oxidative processes of table olives and their nutritional properties. Food Res Int 116:1306–1317. 10.1016/j.foodres.2018.10.020 30716920 10.1016/j.foodres.2018.10.020

[19] International Olive Council (IOC) (2018) Updates series of world statistics on production, imports, exports and consumption. Available online at: https://www.internationaloliveoil.org/wp-content/uploads/2019/11/%20production3_ang.pdf (accessed November 2023)

[20] Hurtado A, Reguant C, Bordons A, Rozès N (2012) Lactic acid bacteria from fermented table olives. Food Microbiol 31:1–8. 10.1016/j.fm.2012.01.006 22475936 10.1016/j.fm.2012.01.006

[21] International Olive Oil Council (IOOC) (2004) The trade standard applying to table olives. Resolution No RES 2/91-IV/04:1–19. https://www.internationaloliveoil.org/wp-content/uploads/2019/11/RES-2-91-IV-04-Eng.pdf (accessed February 2024)

[22] Naureen Z, Bonetti G, Medori MC et al (2022) Foods of the Mediterranean diet: lacto-fermented food, the food pyramid and food combinations. J Prev Med Hyg 63:E28–E35. 10.15167/2421-4248/jpmh2022.63.2S3.2744 36479486 10.15167/2421-4248/jpmh2022.63.2S3.2744 PMC9710393

[23] Şanlier N, Gökcen BB, Sezgin AC (2019) Health benefits of fermented foods. Crit Rev Food Sci Nutr 59:506–527. 10.1080/10408398.2017.1383355 28945458 10.1080/10408398.2017.1383355

[24] Daliu P, Santini A, Novellino E (2019) From pharmaceuticals to nutraceuticals: bridging disease prevention and management. Expert Rev Clin Pharmacol 12:1–7. 10.1080/17512433.2019.1552135 30484336 10.1080/17512433.2019.1552135

[25] Dahiya D, Nigam PS (2022) Probiotics, Prebiotics, Synbiotics, and Fermented Foods as Potential Biotics in Nutrition Improving Health via Microbiome-Gut-Brain Axis. Fermentation 8:303. 10.3390/fermentation8070303

[26] Gibson GR, Hutkins R, Sanders ME et al (2017) Expert Consensus Document: the International Scientific Association for Probiotics and Prebiotics (ISAPP) Consensus Statement on the definition and scope of Prebiotics. Nat Rev Gastroenterol Hepatol 14:491–502. 10.1038/nrgastro.2017.75 28611480 10.1038/nrgastro.2017.75

[27] Hill C, Guarner F, Reid G et al (2014) Expert consensus document: the International Scientific Association for Probiotics and Prebiotics consensus statement on the scope and appropriate use of the term probiotic. Nat Rev Gastroenterol Hepatol 11:506–514. 10.1038/nrgastro.2014.66 24912386 10.1038/nrgastro.2014.66

[28] Shah PN (2007) Functional cultures and health benefits. Int Dairy J 17:1262–1277. 10.1016/j.idairyj.2007.01.014

[29] Soares MB, Martinez RC, Pereira EP et al (2019) The resistance of Bacillus, Bifidobacterium, and Lactobacillus strains with claimed probiotic properties in different food matrices exposed to simulated gastrointestinal tract conditions. Food Res Int 125:108542. 10.1016/j.foodres.2019.108542 31554104 10.1016/j.foodres.2019.108542

[30] Swanson KS, Gibson GR, Hutkins R et al (2020) The International Scientific Association for Probiotics and Prebiotics (ISAPP) Consensus Statement on the definition and scope of Synbiotics. Nat Rev Gastroenterol Hepatol 17:687–701. 10.1038/s41575-020-0344-2 32826966 10.1038/s41575-020-0344-2 PMC7581511

[31] Żółkiewicz J, Marzec A, Ruszczyński M, Feleszko W (2020) Postbiotics-A step beyond pre- and Probiotics. Nutrients 12:2189. 10.3390/nu12082189 32717965 10.3390/nu12082189 PMC7468815

[32] Pluta R, Ułamek-Kozioł M, Januszewski S, Czuczwar SJ (2020) Gut microbiota and pro/prebiotics in Alzheimer’s disease. Aging 12:5539–5550. 10.18632/aging.102930 32191919 10.18632/aging.102930 PMC7138569

[33] Pandey KB, Rizvi SI (2009) Plant polyphenols as dietary antioxidants in human health and disease. Oxid Med Cell Longev 2:270–278. 10.4161/oxim.2.5.9498 20716914 10.4161/oxim.2.5.9498 PMC2835915

[34] Fernández-García E, Carvajal-Lérida I, Pérez-Gálvez A (2009) In vitro bioaccessibility assessment as a prediction tool of nutritional efficiency. Nutr Res 29:751–760. 10.1016/j.nutres.2009.09.016 19932863 10.1016/j.nutres.2009.09.016

[35] Teng H, Chen L (2019) Polyphenols and bioavailability: an update. Crit Rev Food Sci Nutr 59:2040–2051. 10.1080/10408398.2018.1437023 29405736 10.1080/10408398.2018.1437023

[36] Tyssandier V, Lyan B, Borel P (2001) Main factors governing the transfer of carotenoids from emulsion lipid droplets to micelles. BBA Mol Cell Biol L 1533:285–292. 10.1016/S1388-1981(01)00163-9 10.1016/s1388-1981(01)00163-9 11731338

[37] Chitchumroonchokchai C, Schwartz SJ, Failla ML (2004) Assessment of lutein bioavailability from meals and a supplement using simulated digestion and Caco-2 human intestinal cells. J Nutr 134:2280–2286. 10.1093/jn/134.9.2280 15333717 10.1093/jn/134.9.2280

[38] Ryan L, O’connell O, O’Sullivan L, Aherne SA, O’Brien NM (2008) Micellarisation of carotenoids from raw and cooked vegetables. Plant Foods Hum Nutr 63:127–133. 10.1007/s11130-008-0081-0 18587647 10.1007/s11130-008-0081-0

[39] Courraud J, Berger J, Cristol JP, Avallone S (2013) Stability and bioaccessibility of different forms of carotenoids and vitamin A during in vitro digestion. Food Chem 136:871–877. 10.1016/j.foodchem.2012.08.076 23122139 10.1016/j.foodchem.2012.08.076

[40] D’Antuono I, Garbetta A, Ciasca B et al (2016) Biophenols from table olive cv Bella Di Cerignola: chemical characterization, bioaccessibility, and intestinal absorption. J Agric Food Chem 64:5671–5678. 10.1021/acs.jafc.6b01642 27355793 10.1021/acs.jafc.6b01642

[41] Sarcevica I, Hens B, Tomaszewska I, McAllister M (2023) Digitalizing the TIM-1 model using computational approaches–part one: TIM-1 Data Explorer. Mol Pharmaceut 20:5416–5428. 10.1021/acs.molpharmaceut.3c00422 10.1021/acs.molpharmaceut.3c00422 37878746

[42] Popov IV, Koopmans B, Venema K (2024) Modulation of human gut microbiota by linear and branched fructooligosaccharides in an in vitro colon model (TIM-2). J Appl Microbiol 135:lxae170. 10.1093/jambio/lxae170 38986506 10.1093/jambio/lxae170

[43] Zhao D, Zhang K, Guo D, Tong X (2023) Effect of tea polyphenols on the physicochemical, structural and digestive properties of modified high amylose corn starch. Food Funct 14:5196–5204. 10.1039/D2FO04089A 37191069 10.1039/d2fo04089a

[44] Deyaert S, Moens F, Pirovano W, van den Bogert B et al (2023) Development of a reproducible small intestinal microbiota model and its integration into the SHIME^®^-system, a dynamic in vitro gut model. Front Microbiol 13:1054061. 10.3389/fmicb.2022.1054061 37008301 10.3389/fmicb.2022.1054061 PMC10063983

[45] Lessard-Lord J, Lupien‐Meilleur J, Roussel et al (2024) Mathematical modeling of fluid dynamics in in vitro gut fermentation systems: a new tool to improve the interpretation of microbial metabolism. FASEB J 38:e23398. 10.1096/fj.202301739RR 38214938 10.1096/fj.202301739RR

[46] Duque-Soto C, Quintriqueo-Cid A, Rueda-Robles A et al (2022) Evaluation of different Advanced approaches to Simulation of Dynamic in Vitro digestion of polyphenols from different Food Matrices—A. Syst Rev Antioxid 12:101. 10.3390/antiox12010101 10.3390/antiox12010101 PMC9854833 36670962

[47] Wan MLY, Co VA, El-Nezami H (2021) Dietary polyphenol impact on gut health and microbiota. Crit Rev Food Sci Nutr 61:690–711. 10.1080/10408398.2020.1744512 32208932 10.1080/10408398.2020.1744512

[48] Zhang B, Zhang Y, Xing X, Wang S (2022) Health benefits of dietary polyphenols: insight into interindividual variability in absorption and metabolism. Curr Opin Food Sci 48:100941. 10.1016/j.cofs.2022.100941

[49] Wang X, Qi Y, Zheng H (2022) Dietary polyphenol, gut microbiota, and health benefits. Antioxidants 11:1212. 10.3390/antiox11061212 35740109 10.3390/antiox11061212 PMC9220293

[50] Cheng H, Zhang D, Wu J et al (2023) Interactions between gut microbiota and polyphenols: a mechanistic and metabolomic review. Phytomedicine 154979. 10.1016/j.phymed.2023.154979 10.1016/j.phymed.2023.154979 37552899

[51] Kontogianni VG (2014) Novel techniques towards the identification of different classes of polyphenols. In: Watson RR (ed) Polyphenols in plants. Academic, San Diego, pp 159–185. 10.1016/B978-0-12-397934-6.00008-5

[52] Ray SK, Mukherjee S (2021) Evolving interplay between Dietary polyphenols and Gut Microbiota-An Emerging Importance in Healthcare. Front Nutr 8:634944. 10.3389/fnut.2021.634944 34109202 10.3389/fnut.2021.634944 PMC8180580

[53] Gladine C, Rock E, Morand C, Bauchart D, Durand D (2007) Bioavailability and antioxidant capacity of plant extracts rich in polyphenols, given as a single acute dose, in sheep made highly susceptible to lipoperoxidation. Br J Nutr 98:691–701. 10.1017/S0007114507742666 17475083 10.1017/S0007114507742666

[54] Zaripheh S, Erdman JW Jr (2005) The biodistribution of a single oral dose of [14 C]-lycopene in rats prefed either a control or lycopene-enriched diet. J Nutr 135:2212–2218. 10.1093/jn/135.9.2212 16140900 10.1093/jn/135.9.2212

[55] De Boer VC, Dihal AA, van der Woude H et al (2005) Tissue distribution of quercetin in rats and pigs. J Nutr 135:1718–1725. 10.1093/jn/135.7.1718 15987855 10.1093/jn/135.7.1718

[56] Mullen W, Edwards CA, Crozier A (2006) Absorption, excretion and metabolite profiling of methyl-, glucuronyl-, glucosyl- and sulpho-conjugates of quercetin in human plasma and urine after ingestion of onions. Br J Nutr 96:107–116. 10.1079/BJN20061809 16869998 10.1079/bjn20061809

[57] FAO Statistical Database (2009) http://www.faostat.org/. Accessed February 2024

[58] Negro D, Montesano V, Grieco S, Crupi P, Sarli G, De Lisi A, Sonnante G (2012) Polyphenol compounds in artichoke plant tissues and varieties. J Food Sci 77:C244–C252. 10.1111/j.1750-3841.2011.02531.x 22251096 10.1111/j.1750-3841.2011.02531.x

[59] Feiden T, Valduga E, Zeni J, Steffens J (2023) Bioactive compounds from Artichoke and Application potential. Food Technol Biotech 61:312–327. 10.17113/ftb.61.03.23.8038 10.17113/ftb.61.03.23.8038 PMC10666951 38022879

[60] Elsebai MF, Mocan A, Atanasov AG (2016) Cynaropicrin: a comprehensive research review and therapeutic potential as an anti-hepatitis C virus agent. Front Pharmacol 7:472. 10.3389/fphar.2016.00472 28008316 10.3389/fphar.2016.00472 PMC5143615

[61] Rudić S, Dimitrijević-Branković S, Dimitrijević S, Milić M (2021) Valorization of unexploited artichoke leaves dust for obtaining of extracts rich in natural antioxidants. Separ Purif Technol 256:117714. 10.1016/j.seppur.2020.117714

[62] Ceccarelli N, Curadi M, Picciarelli P, Martelloni L, Sbrana C, Giovannetti M (2010) Globe artichoke as a functional food. Mediterr J Nutr Metab 3:197–201. 10.3233/s12349-010-0021-z

[63] Sobh M, Chaouche N, Chaouch A, Echchelh A (2013) Controlled artichoke fermentation by microbial inoculation. BioTechnology: Indian J 7:174–183

[64] Garbetta A, D’Antuono I, Sisto A, Minervini F, Cardinali A, Lavermicocca P (2018) Effect of artichoke fermentation by probiotic strain Lactobacillus paracasei LMG P-22043 and of digestion process on polyphenols and antioxidant activity. J Funct Foods 45:523–529. 10.1016/j.jff.2018.02.020

[65] Lattanzio V, Kroon PA, Linsalata V, Cardinali A (2009) Globe artichoke: a functional food and source of nutraceutical ingredients. J Funct Foods 1:131–144. 10.1016/j.jff.2009.01.002

[66] Sharma P, Verma PK, Pankaj NK, Agarwal S (2021) The phytochemical ingredients and therapeutic potential of *Cynara scolymus* L. Pharm Biom Res. 10.18502/pbr.v7i3.7696

[67] Roberfroid MB (2000) A European consensus of scientific concepts of functional foods. Nutrition 16:689–691. 10.1016/S0899-9007(00)00329-4 10906599 10.1016/s0899-9007(00)00329-4

[68] Sałata A, Lombardo S, Pandino G, Mauromicale G, Buczkowska H, Nurzyńska-Wierdak R (2022) Biomass yield and polyphenol compounds profile in globe artichoke as affected by irrigation frequency and drying temperature. Ind Crop Prod 176:114375. 10.1016/j.indcrop.2021.114375

[69] Garbetta A, Capotorto I, Cardinali A, D’Antuono I, Linsalata V, Pizzi F, Minervini F (2014) Antioxidant activity induced by main polyphenols present in edible artichoke heads: influence of in vitro gastro-intestinal digestion. J Funct Foods 10:456–464. 10.1016/j.jff.2014.07.019

[70] D’Antuono I, Garbetta A, Linsalata V, Minervini F, Cardinali A (2015) Polyphenols from artichoke heads (Cynara cardunculus (L.) subsp. scolymus Hayek): in vitro bio-accessibility, intestinal uptake and bioavailability. Food Funct 6:1268–1277. 10.1039/C5FO00137D 25758164 10.1039/c5fo00137d

[71] Rocchetti G, Giuberti G, Lucchini F, Lucini L (2020) Polyphenols and Sesquiterpene Lactones from Artichoke heads: modulation of starch digestion, Gut Bioaccessibility, and Bioavailability following in vitro digestion and large intestine fermentation. Antioxidants 9:306. 10.3390/antiox9040306 32290151 10.3390/antiox9040306 PMC7222196

[72] Azzini E, Bugianesi R, Romano F et al (2007) Absorption and metabolism of bioactive molecules after oral consumption of cooked edible heads of Cynara scolymus L. (Cultivar Violetto Di Provenza) in human subjects: a pilot study. Br J Nutr 97:963–969. 10.1017/S0007114507617218 17408528 10.1017/S0007114507617218

[73] Domínguez-Fernández M, Yang PYT, Ludwig IA, Clifford MN, Cid C, Rodriguez-Mateos A (2022) In vivo study of the bioavailability and metabolic profile of (poly) phenols after sous-vide artichoke consumption. Food Chem 367:130620. 10.1016/j.foodchem.2021.130620 34343812 10.1016/j.foodchem.2021.130620

[74] Pérez Pulido R, Benomar N, Abriouel H, López RL, Cañamero M, Gálvez A (2005) Microbiological study of lactic acid fermentation of caper berries by molecular and culture-dependent methods. Appl Environ Microbiol 71:7872–7879. 10.1128/AEM.71 16332762 10.1128/AEM.71.12.7872-7879.2005 PMC1317326

[75] Aksay O, Selli S, Kelebek H (2021) LC-DAD‐ESI‐MS/MS-based assessment of the bioactive compounds in fresh and fermented caper (*Capparis spinosa*) buds and berries. Food Chem 337:127959. 10.1016/j.foodchem.2020.127959 32916535 10.1016/j.foodchem.2020.127959

[76] Özcan MM, Uslu N (2023) The effect of fermentation with different additives on bioactive compounds, antioxidant activity, phenolic component, fatty acid composition and mineral substance contents of capers fruits. J Food Meas Charact 17:3896–3908. 10.1007/s11694-023-01909-5

[77] Eddouks M, Maghrani M, Lemhadri A, Ouahidi ML, Jouad H (2002) Ethnopharmacological survey of medicinal plants used for the treatment of diabetes mellitus, hypertension and cardiac diseases in the south-east region of Morocco (Tafilalet). J Ethnopharmacol 82:97–103. 10.1016/s0378-8741(02)00164-2 12241983 10.1016/s0378-8741(02)00164-2

[78] Allaith AAA (2016) Assessment of the antioxidant properties of the caper fruit (Capparis spinosa L.) from Bahrain. J Association Arab Universities Basic Appl Sci 19:1–7. 10.1016/j.jaubas.2014.07.001

[79] Ghafoor K, Al Juhaimi F, Özcan MM, Uslu N, Babiker EE, Mohamed Ahmed IA (2020) Bioactive properties and phenolic compounds in bud, sprout, and fruit of Capparis spp. plants. J Food Process Pres 44:e14357. 10.1111/jfpp.14357

[80] Palomino JM, del Árbol JT, Benomar N, Abriouel H, Cañamero MM, Gálvez A, Pulido RP (2015) Application of Lactobacillus plantarum Lb9 as starter culture in caper berry fermentation. LWT-Food Sci Technol 60:788–794. 10.1016/j.lwt.2014.09.061

[81] Benkerroum N (2013) Traditional fermented foods of north African countries: technology and food safety challenges with regard to microbiological risks. Compr Rev Food Sci F 12:54–89. 10.1111/j.1541-4337.2012.00215.x

[82] Tlili N, Mejri H, Feriani A, Saadaoui E, Khaldi A, Nasri N (2015) Phenolic profile and antioxidant activity of Capparis spinosa seeds harvested from different wild habitats. Ind Crop Prod 76:930–953. 10.1016/j.indcrop.2015.07.040

[83] Ihme N, Kiesewetter H, Jung FA et al (1996) Leg oedema protection from a buckwheat herb tea in patients with chronic venous insufficiency: a single-centre, randomised, double-blind, placebo-controlled clinical trial. Eur J Clin Pharmacol 50:443–447. 10.1007/s002280050138 8858269 10.1007/s002280050138

[84] Grimalt M, Sánchez-Rodríguez L, Hernández F et al (2021) Volatile profile in different aerial parts of two caper cultivars (Capparis spinosa L). J Food Qual 2021:6620776. 10.1155/2021/6620776

[85] Callewaert R, Hugas M, De Vuyst L (2000) Competitiveness and bacteriocin production of enterococci in the production of spanish-style dry fermented sausages. Int J Food Microbiol 57:33–42. 10.1016/S0168-1605(00)00228-2

[86] Francesca N, Barbera M, Martorana A et al (2016) Optimised method for the analysis of phenolic compounds from caper (Capparis spinosa L.) berries and monitoring of their changes during fermentation. Food Chem 196:1172–1179. 10.1016/j.foodchem.2015.10.045 26593604 10.1016/j.foodchem.2015.10.045

[87] Sonmezdag AS, Kelebek H, Selli S (2019) Characterization of aroma-active compounds, phenolics, and antioxidant properties in fresh and fermented capers (Capparis spinosa) by GC‐MS‐olfactometry and LC‐DAD‐ESI‐MS/MS. J Food Sci 84:2449–2457. 10.1111/1750-3841.14777 31476250 10.1111/1750-3841.14777

[88] Jimenez-Lopez J, Ruiz-Medina A, Ortega-Barrales P, Llorent-Martinez EJ (2018) Phytochemical profile and antioxidant activity of caper berries (Capparis spinosa L.): evaluation of the influence of the fermentation process. Food Chem 250:54–59. 10.1016/j.foodchem.2018.01.010 29412927 10.1016/j.foodchem.2018.01.010

[89] Errachidi F, Bour A, Chabir R (2019) Characterization of Moroccan raw and processed caper berries. Mater Today-Proc 13:841–849. 10.1016/j.matpr.2019.04.047

[90] Özcan MM, Uslu N (2023) Effect of different fermentation conditions on bioactive properties, phenolic component and sensory properties of caper (Capparis ovata desf. Var. Ovata) buds. Food Hum 1:553–561. 10.1016/j.foohum.2023.06.031

[91] Arslan D, Ozcan MM (2007) Effect of some organic acids, yoghurt, starter culture and bud sizes on the chemical properties of pickled caper buds. J Food Sci Technol 44:66–69

[92] Annaz H, Sane Y, Bitchagno GTM et al (2022) Caper (Capparis spinosa L.): an updated review on its phytochemistry, nutritional value, traditional uses, and therapeutic potential. Front Pharmacol 13:878749. 10.3389/fphar.2022.878749 35935860 10.3389/fphar.2022.878749 PMC9353632

[93] Bhoyar MS, Mishra GP, Naik PK, Singh SB (2018) Evaluation of antioxidant capacities and total polyphenols in various edible parts of Capparis spinosa L. collected from trans-himalayas. Defe Life Sci J 3:140–145. 10.14429/DLSJ.3.12570

[94] Grimalt M, Almansa MS, Amorós A, García S, Legua P, Hernández F (2019) Antioxidant activity and total phenols in capers (*Capparis spinosa*). Acta Hortic 1254:311–316. 10.17660/ActaHortic.2019.1254.46

[95] Lo Bosco F, Guarrasi V, Moschetti M, Germana MA, Butera D, Corana F, Papetti A (2019) Nutraceutical value of pantelleria capers (*Capparis spinosa* L). J Food Sci 84:2337–2346. 10.1111/1750-3841.14718 31294468 10.1111/1750-3841.14718

[96] Berkel Kaşıkçı M, Bağdatlıoğlu N (2024) Bioaccessibility of phenolic compounds and antioxidant activity in raw and pickled capers. J Food Sci Technol 61:106–116. 10.1007/s13197-023-05824-x 38192703 10.1007/s13197-023-05824-x PMC10771397

[97] Wojdyło A, Nowicka P, Grimalt M et al (2019) Polyphenol compounds and biological activity of caper (*Capparis spinosa* L.) flowers buds. Plants 8:539. 10.3390/plants8120539 31775254 10.3390/plants8120539 PMC6963175

[98] Siracusa L, Kulisic-Bilusic T, Politeo O et al (2011) Phenolic composition and antioxidant activity of aqueous infusions from Capparis spinosa L. and Crithmum Maritimum L. before and after submission to a two-step in vitro digestion model. J Agric Food Chem 59:12453–12459. 10.1021/jf203096q 22017607 10.1021/jf203096q

[99] Tayiroglu B, Incedayi B (2021) Nutritional potential characterization and bioactive properties of caper products. J Food Process Preserv 45:e14670. 10.1111/jfpp.14670

[100] Boskou D, Camposeo S, Clodoveo ML (2015) Table olives as sources of bioactive compounds. In: Boskou D (ed) Olive and olive oil bioactive constituents. AOCS, Urbana, IL, USA, pp 217–259. 10.1016/B978-1-63067-041-2.50014-8

[101] Brenes M, Romero C, García P, Garrido A (2004) Absorption of sorbic and benzoic acids in the flesh of table olives. Eur Food Res Technol 219:75–79. 10.1007/s00217-004-0893-6

[102] Hurtado A, Reguant C, Esteve-Zarzoso B, Bordons A, Rozès N (2008) Microbial population dynamics during the processing of Aberquina table olives. Food Res Int 41:738–744. 10.1016/j.foodres.2008.05.007

[103] Servili M, Settanni L, Veneziani G et al (2006) The use of Lactobacillus pentosus 1MO to shorten the debittering process time of black table olives (cv. Itrana and Leccino): a pilot-scale application. J Agric Food Chem 54:3869–3875. 10.1021/jf053206y 16719508 10.1021/jf053206y

[104] Sabatini N, Marsilio V (2008) Volatile compounds in table olives (Olea Europaea L., Nocellara Del Belice cultivar). Food Chem 107:1522–1528. 10.1016/j.foodchem.2007.10.008

[105] Tufariello M, Durante M, Ramires FA et al (2015) New process for production of fermented black table olives using selected autochthonous microbial resources. Front Microbiol 6:1007. 10.3389/fmicb.2015.01007 26441932 10.3389/fmicb.2015.01007 PMC4585182

[106] Caggia C, Randazzo CL, Di Salvo M, Romeo F, Giudici P (2004) Occurrence of Listeria monocytogenes in green table olives. J Food Prot 67:2189–2194. 10.4315/0362-028X-67.10.2189 15508629 10.4315/0362-028x-67.10.2189

[107] Cawthorne A, Celentano LP, D’Ancona F, Bella A, Massari M, Anniballi F, Salmaso S (2005) Botulism and preserved green olives. Emerg Infect Dis 11:781. 10.3201/eid1105.041088 15898180 10.3201/eid1105.041088 PMC3320370

[108] Sánchez AH, De Castro A, Rejano L, Montaño A (2000) Comparative study on chemical changes in olive juice and brine during green olive fermentation. J Agric Food Chem 48:5975–5980. 10.1021/jf000563u 11141267 10.1021/jf000563u

[109] Tarantini A, Crupi P, Ramires FA et al (2024) Study of the effects of pasteurization and selected microbial starters on functional traits of fermented table olives. Food Microbiol 122:104537. 10.1016/j.fm.2024.104537 38839217 10.1016/j.fm.2024.104537

[110] Nisiotou AA, Chorianopoulos N, Nychas GJ, Panagou EZ (2010) Yeast heterogeneity during spontaneous fermentation of black Conservolea olives in different brine solutions. J Appl Microbiol 108:396–405. 10.1111/j.1365-2672.2009.04424.x 20438554 10.1111/j.1365-2672.2009.04424.x

[111] Bautista-Gallego J, Rodríguez-Gómez F, Barrio E, Querol A, Garrido-Fernández A, Arroyo-López FN (2011) Exploring the yeast biodiversity of green table olive industrial fermentations for technological applications. Int J Food Microbiol 147:89–96. 10.1016/j.ijfoodmicro.2011.03.013 21497408 10.1016/j.ijfoodmicro.2011.03.013

[112] Psani M, Kotzekidou P (2006) Technological characteristics of yeast strains and their potential as starter adjuncts in greek-style black olive fermentation. World J Microbiol Biotechnol 22:1329–1336. 10.1007/s11274-006-9180-y

[113] Chytiri A, Tasioula-Margari M, Bleve G, Kontogianni VG, Kallimanis A, Kontominas MG (2020) Effect of different inoculation strategies of selected yeast and LAB cultures on Conservolea and Kalamàta table olives considering phenol content, texture, and sensory attributes. J Sci Food Agric 100:926–935. 10.1002/jsfa.10019 31523827 10.1002/jsfa.10019

[114] Vaccalluzzo A, Pino A, Russo N, De Angelis M, Caggia C, Randazzo CL (2020) FoodOmics as a new frontier to reveal microbial community and metabolic processes occurring on table olives fermentation. Food Microbiol 92:103606. 10.1016/j.fm.2020.103606 32950142 10.1016/j.fm.2020.103606

[115] Brenes M, Kailis SG (2021) Naturally processed table olives, their preservation and uses. In: Olives and olive oil in health and disease prevention. Academic Press, pp 15–25. 10.1016/B978-0-12-819528-4.00054-7

[116] Arfaoui L (2021) Dietary plant polyphenols: effects of Food Processing on their content and bioavailability. Molecules 26:2959. 10.3390/molecules26102959 34065743 10.3390/molecules26102959 PMC8156030

[117] D’Antuono I, Bruno A, Linsalata V et al (2018) Fermented apulian table olives: Effect of selected microbial starters on polyphenols composition, antioxidant activities and bioaccessibility. Food Chem 248:137–145. 10.1016/j.foodchem.2017.12.032 29329836 10.1016/j.foodchem.2017.12.032

[118] Reboredo-Rodríguez P, González-Barreiro C, Cancho-Grande B, Fregapane G, Salvador MD, Simal-Gándara J (2015) Characterisation of extra virgin olive oils from galician autochthonous varieties and their co-crushings with Arbequina and Picual Cv. Food Chem 176:493–503. 10.1016/j.foodchem.2014.12.078 25624261 10.1016/j.foodchem.2014.12.078

[119] Gebhardt R, Fausel M (1997) Antioxidant and hepatoprotective effects of artichoke extracts and constituents in cultured rat hepatocytes. Toxicol vitro 11:669–672. 10.1016/S0887-2333(97)00078-7 10.1016/s0887-2333(97)00078-7 20654368

[120] Jimenez-Escrig A, Dragsted LO, Daneshvar B, Pulido R, Saura-Calixto F (2003) In vitro antioxidant activities of edible artichoke (Cynara scolymus L.) and effect on biomarkers of antioxidants in rats. J Agr Food Chem 51:5540–5545. 10.1021/jf030047e 12926911 10.1021/jf030047e

[121] Pérez-García F, Adzet T, Cañigueral S (2000) Activity of artichoke leaf extract on reactive oxygen species in human leukocytes. Free Radical Res 33:661–665. 10.1080/10715760000301171 11200096 10.1080/10715760000301171

[122] Iglesias-Carres L, Bruno A, D’Antuono I, Linsalata V, Cardinali A, Neilson AP (2023) In vitro evidences of the globe artichoke antioxidant, cardioprotective and neuroprotective effects. J Funct Foods 107:105674. 10.1016/j.jff.2023.105674

[123] D’Antuono I, Carola A, Sena LM, Linsalata V, Cardinali A, Logrieco AF, Colucci MG, Apone F (2018) Artichoke polyphenols produce skin anti-age effects by improving endothelial cell integrity and functionality. Molecules 23:2729. 10.3390/molecules23112729 30360471 10.3390/molecules23112729 PMC6278506

[124] Rodriguez TS, Giménez DG, De la Puerta Vázquez R (2002) Choleretic activity and biliary elimination of lipids and bile acids induced by an artichoke leaf extract in rats. Phytomedicine 9:687–693. 10.1078/094471102321621278 12587687 10.1078/094471102321621278

[125] Gebhardt R (1998) Inhibition of cholesterol biosynthesis in primary cultured rat hepatocytes by artichoke (Cynara scolymus L.) extracts. J Pharmacol Exp Ther 286:1122–1128 9732368

[126] Kraft K (1997) Artichoke leaf extract-recent findings reflecting effects on lipid metabolism, liver and gastrointestinal tracts. Phytomedicine 4:369–378. 10.1016/S0944-7113(97)80049-9 23195590 10.1016/S0944-7113(97)80049-9

[127] Brown JE, Rice-Evans CA (1998) Luteolin-rich artichoke extracts protect low density lipoprotein from oxidation in vitro. Free Radic Res 29:247–255. 10.1080/10715769800300281 9802556 10.1080/10715769800300281

[128] Li H, Xia N, Brausch I, Yao Y, Fo¨ rstermann U (2004) Flavonoids from artichoke (Cynara scolymus L.) up-regulate endothelial-type nitric oxide synthase gene expression in human endothelial cells. J Pharmacol Exp Ther 310:926–932. 10.1124/jpet.104.066639 15123766 10.1124/jpet.104.066639

[129] Aliyazicioglu R, Eyupoglu OE, Sahin H, Yildiz O, Baltas N (2013) Phenolic components, antioxidant activity, and mineral analysis of Capparis spinosa L. Afr J Biotechnol 12:6643–6649. 10.5897/AJB2013.13241

[130] Mehrzadi S, Mirzaei R, Heydari M, Sasani M, Yaqoobvand B, Huseini HF (2021) Efficacy and safety of a traditional herbal combination in patients with type II diabetes mellitus: a randomized controlled trial. J Diet Suppl 18:31–43. 10.1080/19390211.2020.1727076 32081056 10.1080/19390211.2020.1727076

[131] Sher M, Alyemeni MN (2010) Ethnobotanical and pharmaceutical evaluation of Capparis spinosa L, validity of local folk and Unani system of medicine. J Med Plants Res 4:1751–1756. 10.9734/MRJI/2021/v31i130297

[132] Aichour R, Benzidane N, Arrar L, Charef N, Baghiani A (2018) Hepatoprotective and anti-inflammatory activities of Algerian Capparis spinosa. L. Annu Res Rev Bio 25:1–12. 10.9734/arrb/2018/40410

[133] Mohebali N, Shahzadeh Fazeli SA, Ghafoori H et al (2018) Effect of flavonoids Rich Extract of Capparis Spinosa on Inflammatory involved genes in amyloid-Beta peptide injected rat model of Alzheimer’s Disease. Nutr Neurosci 21:143–150. 10.1080/1028415X.2016.1238026 27778760 10.1080/1028415X.2016.1238026

[134] Kulisic-Bilusic T, Schmöller I, Schnäbele K, Siracusa L, Ruberto G (2012) The anticarcinogenic potential of essential oil and aqueous infusion from caper (Capparis spinosa L). Food Chem 132:261–267. 10.1016/j.foodchem.2011.10.074 26434289 10.1016/j.foodchem.2011.10.074

[135] Adwan GM, Omar GI (2021) Evaluation of antimicrobial activity and genotoxic potential of Capparis spinosa (L.) plant extracts. Microbiol Res J Int 31:48–57. 10.9734/mrji/2021/v31i130297

[136] Gull T, Sultana B, Bhatti IA, Jamil A (2015) Antibacterial potential of Capparis Spinosa and Capparis Decidua extracts. Int J Adv Biol 17:727–733. 10.17957/ijab/14.0007

[137] Andrikopoulos NK, Kaliora AC, Assimopoulou AN, Papageorgiou VP (2002) Inhibitory activity of minor polyphenolic and nonpolyphenolic constituents of olive oil against in vitro low-density lipoprotein oxidation. J Med Food 5:1–7. 10.1089/109662002753723160 12511107 10.1089/109662002753723160

[138] Arts IC, Hollman PC (2005) Polyphenols and disease risk in epidemiologic studies. Am J Clin Nutr 81:317S-325S. 10.1093/ajcn/81.1.317S 10.1093/ajcn/81.1.317S 15640497

[139] Pignatelli P, Pulcinelli FM, Lenti L, Paolo Gazzaniga P, Violi F (1998) Hydrogen peroxide is involved in collagen-induced platelet activation. Blood 91:484–490. 10.1182/blood.V91.2.484 9427701

[140] Somova LI, Shode FO, Ramnanan P, Nadar A (2003) Antihypertensive, antiatherosclerotic and antioxidant activity of triterpenoids isolated from Olea europaea, subspecies africana leaves. J Ethnopharmacol 84:299–305. 10.1016/S0378-8741(02)00332-X 12648829 10.1016/s0378-8741(02)00332-x

[141] Al-Azzawie HF, Alhamdani MSS (2006) Hypoglycemic and antioxidant effect of oleuropein in alloxan-diabetic rabbits. Life Sci 78:1371–1377. 10.1016/j.lfs.2005.07.029 16236331 10.1016/j.lfs.2005.07.029

[142] Rodriguez-Rodriguez R, Herrera MD, De Sotomayor MA, Ruiz-Gutierrez V (2009) Effects of pomace olive oil-enriched diets on endothelial function of small mesenteric arteries from spontaneously hypertensive rats. Brit J Nutr 102:1435–1444. 10.1017/S0007114509990754 19563692 10.1017/S0007114509990754

[143] Hamdi HK, Castellon R (2005) Oleuropein, a non-toxic olive iridoid, is an anti-tumor agent and cytoskeleton disruptor. Biochem Bioph Res Co 334:769–778. 10.1016/j.bbrc.2005.06.161 10.1016/j.bbrc.2005.06.161 16024000

[144] Pawlowska E, Szczepanska J, Koskela A, Kaarniranta K, Blasiak J (2019) Dietary polyphenols in age-related macular degeneration: protection against oxidative stress and beyond. Oxid Med Cell Longev 2019:1–13. 10.1155/2019/9682318 10.1155/2019/9682318 PMC6451822 31019656

[145] Russo GL, Spagnuolo C, Russo M, Tedesco I, Moccia S, Cervellera C (2020) Mechanisms of aging and potential role of selected polyphenols in extending healthspan. Biochem Pharmacol 173:113719. 10.1016/j.bcp.2019.113719 31759977 10.1016/j.bcp.2019.113719

[146] Abolaji AO, Adedara AO, Adie MA, Vicente-Crespo M, Farombi EO (2018) Resveratrol prolongs lifespan and improves 1-methyl-4-phenyl-1,2,3,6-tetrahydropyridine-induced oxidative damage and behavioural deficits in *Drosophila melanogaster*. Biochem Bioph Res Co 503:1042–1048. 10.1016/j.bbrc.2018.06.114 10.1016/j.bbrc.2018.06.114 29935183

[147] Terracina S, Petrella C, Francati S et al (2022) Antioxidant intervention to improve cognition in the aging brain: the example of hydroxytyrosol and resveratrol. Int J Mol Sci 23:15674. 10.3390/ijms232415674 36555317 10.3390/ijms232415674 PMC9778814

[148] Wu YT, Lin LC, Tsai TH (2009) Measurement of free hydroxytyrosol in microdialysates from blood and brain of anesthetized rats by liquid chromatography with fluorescence detection. J Chromatogr A 1216:3501–3507. 10.1016/j.chroma.2008.10.116 19022451 10.1016/j.chroma.2008.10.116

[149] D’Andrea G, Ceccarelli M, Bernini R, Clemente M, Santi L, Caruso C, Micheli L, Tirone F (2020) Hydroxytyrosol stimulates neurogenesis in aged dentate gyrus by enhancing stem and progenitor cell proliferation and neuron survival. FASEB J 34:4512–4526. 10.1096/fj.201902643R 32027412 10.1096/fj.201902643R

[150] Micheli L, Bertini L, Bonato A, Villanova N, Caruso C, Caruso M, Bernini R, Tirone F (2023) Role of Hydroxytyrosol and Oleuropein in the Prevention of Aging and Related disorders: Focus on Neurodegeneration, skeletal muscle dysfunction and gut microbiota. Nutrients 15:1767. 10.3390/nu15071767 37049607 10.3390/nu15071767 PMC10096778

[151] Abd El-Aziz NM, Awad OME, Shehata MG, El-Sohaimy SA (2021) Antioxidant and anti-acetylcholinesterase potential of artichoke phenolic compounds. Food Biosci 41:101006. 10.1016/j.fbio.2021.101006

[152] Brimijoin S, Chen VP, Pang YP, Geng L, Gao Y (2016) Physiological roles for butyrylcholinesterase: a BChE-Ghrelin. Axis Chem Biol Interact 259:271–275. 10.1016/j.cbi.2016.02.013 26915976 10.1016/j.cbi.2016.02.013 PMC4995144

[153] Granato D, Branco GF, Nazzaro F, Cruz AG, Faria JA (2010) Functional foods and nondairy probiotic food development: trends, concepts, and products. Compr Rev Food Sci Food Saf 9:292–302. 10.1111/j.1541-4337.2010.00110.x 33467814 10.1111/j.1541-4337.2010.00110.x

[154] Kabir SML, Islam SS, Tuhin-Al-Ferdous, Akhter AHMT (2023) Production, cost analysis, and marketing of Probiotics. In: Amaresan N, Dharumadurai D, Babalola OO (eds) Food Microbiology based entrepreneurship. Springer, Singapore. 10.1007/978-981-19-5041-4_16

[155] De Bellis P, Sisto A, Lavermicocca P (2021) Probiotic bacteria and plant-based matrices: an association with improved health-promoting features. J Funct Foods 87:104821. 10.1016/j.jff.2021.104821

[156] Pourjafar H, Pimentel TC, Baú TR (2023) Probiotic fermented vegetables. In: Gomes da Cruz, A., Silva, M.C., Colombo Pimentel, T., Esmerino, E.A., Verruck, S. (eds) Probiotic Foods and Beverages. Methods and Protocols in Food Science. Humana, New York, NY. 10.1007/978-1-0716-3187-4_8

[157] Kumar S, Rattu G, Mitharwal S et al (2022) Trends in non-dairy‐based probiotic food products: advances and challenges. J Food Process Preserv 46:e16578. 10.1111/jfpp.16578

[158] Bustos AY, Font G, Taranto MP (2023) Fruit and vegetable snacks as carriers of probiotics and bioactive compounds: a review. Int J Food Sci Tech 58:3211–3223. 10.1111/ijfs.16400

[159] Rahman MS, Emon DD, Toma MA et al (2023) Recent advances in probiotication of fruit and vegetable juices. J Adv Vet Anim Res 10:522–537. 10.5455/javar.2023.j706 37969792 10.5455/javar.2023.j706 PMC10636081

[160] Lavermicocca P, Valerio F, Lonigro SL et al (2005) Study of adhesion and survival of lactobacilli and bifidobacteria on table olives with the aim of formulating a new probiotic food. App Environ Microb 71:4233–4240. 10.1128/AEM.71.8.4233-4240.2005 10.1128/AEM.71.8.4233-4240.2005 PMC1183302 16085808

[161] Valerio F, De Bellis P, Lonigro SL, Morelli L, Visconti A, Lavermicocca P (2006) In vitro and in vivo survival and transit tolerance of potentially probiotic strains carried by artichokes in the gastrointestinal tract. App Environ Microb 72:3042–3045. 10.1128/AEM.72.4.3042-3045.2006 10.1128/AEM.72.4.3042-3045.2006 PMC1449069 16598015

[162] Mariappan B, Prakash S, Binesh A (2023) Probiotic nanoparticles for food. In: Jyothis Mathew, Midhun Sebastian Jose, Radhakrishnan E.K., Ajay Kumar (eds) Recent advances in aquaculture microbial technology. Academic Press, pp 307–338. 10.1016/B978-0-323-90261-8.00008-0

[163] Neekhra S, Pandith JA, Mir NA, Manzoor A, Ahmad S, Ahmad R, Sheikh RA (2022) Innovative approaches for microencapsulating bioactive compounds and probiotics: an updated review. J Food Process Pres 46:e16935. 10.1111/jfpp.16935

[164] Valerio F, Lonigro SL, Giribaldi M, Di Biase M, De Bellis P, Cavallarin L, Lavermicocca P (2015) Probiotic Lactobacillus paracasei IMPC 2.1 strain delivered by ready-to-eat swordfish fillets colonizes the human gut after alternate-day supplementation. J Funct Foods 17:468–475. 10.1016/j.jff.2015.05.044

[165] Garcia-Henao CE, Valderrama‐Sanchez V, Arboleda‐Murillo JA, Pinzon MI, Sanchez LT, Villa CC (2023) Bioactive food coating: a review. Packag Technol Sci 36:3–13. 10.1002/pts.2689

[166] Valerio F, Volpe MG, Santagata G, Boscaino F, Barbarisi C, Di Biase M, Bavaro AR, Lonigro SL, Lavermicocca P (2020) The viability of probiotic Lactobacillus paracasei IMPC2.1 coating on apple slices during dehydration and simulated gastro-intestinal digestion. Food Biosci 34:100533. 10.1016/j.fbio.2020.100533

[167] Oliveira AS, Niro CM, Bresolin JD, Soares VF, Ferreira MD, Sivieri K, Azeredo HM (2021) Dehydrated strawberries for probiotic delivery: influence of dehydration and probiotic incorporation methods. LWT 144:111105. 10.1016/j.lwt.2021.111105

[168] Cordenunsi BR, Oliveira do Nascimento JR, Genovese MI, Lajolo FM (2002) Influence of cultivar on quality parameters and chemical composition of strawberry fruits grown in Brazil. J Agr Food Chem 50:2581–2586. 10.1021/jf011421i 11958626 10.1021/jf011421i

[169] Vivek K, Mishra S, Pradhan RC (2022) A comprehensive review on microencapsulation of probiotics: technology, carriers and current trends. App Food Res 100248. 10.1016/j.afres.2022.100248

[170] Sarvan I, Valerio F, Lonigro SL, de Candia S, Verkerk R, Dekker M, Lavermicocca P (2013) Glucosinolate content of blanched cabbage (Brassica oleracea var. capitata) fermented by the probiotic strain *Lactobacillus paracasei* LMG-P22043. Food Res Int 54:706–710. 10.1016/j.foodres.2013.07.065

[171] Pimentel TC, Da Costa WKA, Barão CE, Rosset M, Magnani M (2021) Vegan probiotic products: a modern tendency or the newest challenge in functional foods. Food Res Int 140:110033. 10.1016/j.foodres.2020.110033 33648260 10.1016/j.foodres.2020.110033

[172] Sireswar S, Dey G, Biswas S (2021) Influence of fruit-based beverages on efficacy of *lacticaseibacillus rhamnosus* GG (*Lactobacillus rhamnosus* GG) against DSS-induced intestinal inflammation. Food Res Int 149:110661. 10.1016/j.foodres.2021.110661 34600663 10.1016/j.foodres.2021.110661

[173] Siripun P, Chaiyasut C, Lailerd N, Makhamrueang N, Kaewarsar E, Sirilun S (2022) A pilot study of whether or not vegetable and fruit juice containing *Lactobacillus paracasei* lowers blood lipid levels and oxidative stress markers in Thai patients with dyslipidemia: a randomized controlled clinical trial. App Sci 12:4913. 10.3390/app12104913

[174] Cardelo MP, Corina A, Leon-Acuña A et al (2022) Effect of the Mediterranean diet and probiotic supplementation in the management of mild cognitive impairment: Rationale, methods, and baseline characteristics. Front Nutr 9:1037842. 10.3389/fnut.2022.1037842 36570150 10.3389/fnut.2022.1037842 PMC9773830

[175] Riezzo G, Orlando A, D’Attoma B, Guerra V, Valerio F, Lavermicocca P, De Candia S, Russo F (2012) Randomised clinical trial: efficacy of Lactobacillus paracasei enriched artichokes in the treatment of patients with functional constipation - A double-blind, controlled, crossover study. Aliment Pharm Th 35:441–450. 10.1111/j.1365-2036.2011.04970.x 10.1111/j.1365-2036.2011.04970.x 22225544

[176] Valerio F, De Candia S, Lonigro SL et al (2011) Role of the probiotic strain *Lactobacillus paracasei* LMGP22043 carried by artichokes in influencing faecal bacteria and biochemical parameters in human subjects. J Appl Microbiol 111:155–164. 10.1111/j.1365-2672.2011.05019.x 21447019 10.1111/j.1365-2672.2011.05019.x

[177] Dimidi E, Christodoulides S, Fragkos KC, Scott SM, Whelan K (2014) The effect of probiotics on functional constipation in adults: a systematic review and meta-analysis of randomized controlled trials. Am J Clin Nutr 100:1075–1084. 10.3945/ajcn.114.089151 25099542 10.3945/ajcn.114.089151

[178] Genevois M, Escalada Pla S, Flores S (2017) Novel strategies for fortifying vegetable matrices with iron and Lactobacillus casei simultaneously. Food Sci Tech- LWT 79:34–41. 10.1016/j.lwt.2017.01.019

[179] Argyri AA, Nisiotou AA, Mallouchos A, Panagou EZ, Tassou CC (2014) Performance of two potential probiotic Lactobacillus strains from the olive microbiota as starters in the fermentation of heat shocked green olives. Int J Food Microbiol 171:68–76. 10.1016/j.ijfoodmicro.2013.11.003 24334091 10.1016/j.ijfoodmicro.2013.11.003

[180] Rodríguez-Gόmez F, Romero-Gil V, Arroyo-Lόpez FN et al (2017) Assessing the challenges in the application of potential probiotic lactic acid bacteria in the large-scale fermentation of Spanish-style table olives. Article 915 Front Microbiol 8. 10.3389/fmicb.2017.00915 10.3389/fmicb.2017.00915 PMC5434132 28567038

[181] Alves M, Peres CM, Hernandez-Mendonza A, Bronze MR, Peres C, Malcata FX (2015) Olive paste as vehicle for delivery of potential probiotic Lactobacillus plantarum 33. Food Res Int 75:61–70. 10.1016/j.foodres.2015.04.048 28454973 10.1016/j.foodres.2015.04.048

[182] Beganovíc J, Pavunc AL, Gjuraci´c K, ˇSpoljarec M, ˇSuˇskovi´c J, Kos B (2011) Improved sauerkraut production with probiotic strain Lactobacillus plantarum L4 and Leuconostoc mesenteroides LMG 7954. J Food Sci 76:M124–M129. 10.1111/j.1750-3841.2010.02030.x 21535775 10.1111/j.1750-3841.2010.02030.x

[183] Shigematsu E, Dorta C, Rodrigues FJ, Cedran MF, Giannoni JA, Oshiiwa M, Mauro MA (2018) Edible coating with probiotic as a quality factor for minimally processed carrots. J Food Sci Tech 55:3712–3720. 10.1007/s13197-018-3301-0 10.1007/s13197-018-3301-0 PMC6098787 30150831

[184] Barbu V, Cotârleț M, Bolea CA, Cantaragiu A, Andronoiu DG, Bahrim GE, Enachi E (2020) Three types of beetroot products enriched with lactic acid bacteria. Foods 9:786. 10.3390/foods9060786 32545898 10.3390/foods9060786 PMC7353617

